# Psychometric properties of the Arabic Perceived Digital Well-Being Scale (PDWS) in Saudi young adults

**DOI:** 10.1371/journal.pone.0354474

**Published:** 2026-07-30

**Authors:** Emadeldin M. Elsokkary

**Affiliations:** Department of Psychology, College of Social Sciences, Imam Mohammad Ibn Saud Islamic University (IMSIU), Riyadh, Saudi Arabia; Catholic University of Pernambuco: Universidade Catolica de Pernambuco, BRAZIL

## Abstract

Rapid digital transformation in Saudi Arabia has intensified young adults’ day-to-day reliance on smartphones, increasing the need for balanced, culturally appropriate measures of perceived digital well-being. This study aimed to psychometrically validate the Arabic version of the Perceived Digital Well-Being Scale (PDWS) among Saudi young adults aged (18–25). A cross-sectional instrument-validation study was conducted with (N = 450) participants. The full sample was randomly split into two independent analytic subsamples: Sample (1) for exploratory factor analysis (EFA) (n = 225) and Sample (2) for confirmatory factor analysis (CFA) (n = 225); convergent validity was examined in both subsamples. EFA supported a clear three-factor structure (Emotional, Social, Cognitive). CFA confirmed a second-order model with excellent fit: χ²(116)=133.275, p = .130, χ²/df=(1.149), CFI=(.986), TLI=(.983), RMSEA=(.026), and SRMR=(.055). Convergent validity was supported by positive associations with life satisfaction (SWLS) and negative associations with fear of missing out (FoMO), with the same pattern observed across both subsamples (Sample (2): SWLS r=(.416–.550), p < .01; FoMO r=(−.321–-.254), p < .01). Internal consistency was acceptable across subsamples (Cronbach’s α=(.759–.886); McDonald’s ω=(.761–.889)). Overall, the Arabic PDWS demonstrates strong initial evidence of structural validity, theoretically consistent convergent validity, and adequate reliability, supporting its use as a multidimensional measure of perceived smartphone-related digital well-being among Saudi emerging adults.

## Introduction

Digital technology has become the default setting for how many young people learn, socialize, and manage daily life. In Saudi Arabia, this shift has unfolded alongside rapid national digital transformation and widespread adoption of smartphones and social platforms, making digitally mediated routines especially salient in the lives of young adults. Yet the psychological significance of this environment is still not fully captured by much of the existing literature, which has often been dominated by risk-focused outcomes such as problematic smartphone use and addiction-like patterns [1]. What remains comparatively underdeveloped—particularly in non-Western settings—is a balanced, person-centered understanding of digital well-being: how individuals themselves evaluate whether their digital engagement supports or undermines their functioning [2–5].

Digital adoption indicators support the relevance of this focus in Saudi Arabia. Recent national and international reports indicate that internet penetration in Saudi Arabia has reached approximately (99%), with mobile phones accounting for (99.4%) of internet browsing device use [6]. DataReportal further reported (33.9) million internet users in Saudi Arabia at the start of (2025), alongside (48.1) million cellular mobile connections, equivalent to (140%) of the total population, reflecting that individuals may hold multiple mobile connections. Social media use is similarly extensive: Saudi Arabia had (34.1) million social media user identities in January (2025), equivalent to (99.6%) of the total population, a level substantially above the global social media user-identity penetration of (63.9%) [7,8]. Together, these indicators show that Saudi Arabia represents a highly connected digital context in which smartphone-based and socially mediated experiences are deeply embedded in everyday life.

A major reason for this gap is conceptual and measurement inconsistency. “Digital well-being” has been used to refer to very different ideas—ranging from time-management and self-control to emotional strain, social connection, cognitive overload, and perceived life balance [2–5]. In practice, simply quantifying exposure (e.g., hours online) rarely tells us whether technology is experienced as helpful or harmful in daily life, and evidence suggests that links between digital engagement and well-being are often small on average and can vary across people, contexts, and developmental periods [9–12]. A more informative approach is to assess the perceived impact of digital engagement on key domains of functioning. This perspective aligns with recent measurement work that treats digital well-being as a subjective appraisal of digital life across emotional, social, and cognitive spheres [13].

The Saudi context strengthens the case for such an approach. Social life is strongly relationship-oriented, and digital communication is woven into everyday obligations and expectations. In settings where relational closeness and responsiveness carry social meaning, technology can become a source of pressure rather than convenience: messages can imply an expectation of timely replies, group chats can create continuous social “presence,” and delays in response may be interpreted as disengagement. These culturally shaped dynamics can turn everyday connectivity into a subtle burden—especially for young adults navigating social identity, peer evaluation, and boundary-setting. In other words, the same tools that facilitate closeness may also intensify social demand, thereby shaping how digital engagement is appraised in terms of well-being [2,3].

The present study focuses on Saudi young adults aged (18–25). This age range corresponds closely to what Arnett (2000) described as emerging adulthood, a developmental period characterized by identity exploration and intensive negotiation of social roles and relationships [14]. Official Saudi youth statistics define youth broadly as individuals aged (15–34), a category that includes the present study’s target age range, and indicate that this group represents (36.2%) of the Saudi population, while Saudis under the age of (35) represent approximately (71%) of the total Saudi population [15]. The choice of (18–25) also addresses an important methodological and interpretive issue. National youth statistics often use broader categories (e.g., “youth” spanning mid-adolescence through the early thirties), which are valuable for population description but can combine markedly different developmental stages and life contexts. In contrast, concentrating on (18–25) provides a more developmentally coherent group in which digitally mediated life is especially central to peer connection, identity expression, and daily routines—while also reducing age-related heterogeneity that can obscure factor structure and validity patterns in psychometric testing.

Together, these conceptual, cultural, and developmental considerations point to the need for a measure that captures how Saudi young adults themselves appraise the perceived impact of digital engagement on everyday functioning. Against this background, the Perceived Digital Well-Being Scale (PDWS) offers a focused solution to a persistent measurement problem: how to capture the lived balance of benefits and costs in digital life without presuming that technology is uniformly “good” or “bad.” The PDWS is grounded in the idea that digital engagement can simultaneously support connection and productivity while also straining attention, emotions, or social comparison. The scale originates from the Perceived Digital Well-Being in Adolescence Scale (PDWBA), developed by Rosič et al. [13] to assess adolescents’ subjective perceptions of whether the benefits of smartphone use outweigh its drawbacks across emotional, social, and cognitive domains. The original instrument included 17 bipolar items and was developed through a scale-development process involving a multidisciplinary team with expertise in digital media effects, methodology, and adolescent development. This development process provided content-related support for the item pool, while initial psychometric testing supported the internal three-domain structure in Slovenian adolescents. In a recent psychometric validation, Vera Cruz et al. [16] examined an adapted young-adult version of the instrument, renamed the PDWS, in samples from the United States and the United Kingdom. Their findings supported the three-factor structure, internal consistency, convergent validity through theoretically consistent associations with digital flourishing and digital stress, and measurement invariance across national samples, although invariance across gender was limited. These findings are promising, but they do not resolve a central question for research in Arabic-speaking settings: will the same measurement structure and validity evidence hold in Saudi young adults, where cultural norms, communication practices, and digitally mediated social obligations may influence how items are understood and endorsed?

From a psychometric standpoint, scale use in a new linguistic and cultural context requires more than translation. The Standards for Educational and Psychological Testing emphasize that validity is a cumulative argument supported by multiple sources of evidence aligned with intended score interpretations [17]. Similarly, the International Test Commission’s test adaptation guidelines stress careful translation procedures, documentation of decisions, and evaluation of measurement equivalence in the target population [18]. Modern measurement frameworks further highlight that structural validity, reliability, and hypothesis testing should be examined transparently and interpreted in relation to a construct’s expected nomological network [19,20]. These principles are particularly relevant for perceived digital well-being, because culturally patterned expectations about responsiveness, privacy, and social participation can shape both technology behavior and the perceived meaning of that behavior [2–4].

Consistent with these standards, the current study evaluates the PDWS factor structure in Saudi young adults using exploratory and confirmatory factor-analytic evidence. Conceptually, the PDWS comprises correlated first-order domains (emotional, social, and cognitive) that may also reflect a broader higher-order perceived digital well-being factor. Because the PDWS is intended to capture an overarching appraisal of digital life across domains, specifying a second-order CFA model provides a rigorous test of structural validity while remaining faithful to the measure’s conceptual foundation [13,16].

To strengthen validity evidence beyond structure, the present study places PDWS scores within a focused nomological network using two convergent benchmarks: Fear of Missing Out (FoMO) and life satisfaction. Przybylski et al. (2013) defined FoMO as a persistent apprehension that others might be having rewarding experiences from which one is absent, coupled with a desire to stay continually connected [21]. Conceptually, FoMO can push digital engagement toward anxious monitoring, heightened social comparison, and difficulty disengaging—conditions that are likely to be experienced as draining rather than supportive. Evidence syntheses indicate that FoMO is reliably linked to more problematic patterns of social networking use, supporting its relevance as a convergent benchmark [22]. Recent Saudi evidence also supports the local relevance of FoMO and shows meaningful associations with technology-related outcomes [23]. On these grounds, higher FoMO is expected to relate to lower perceived digital well-being.

Life satisfaction provides a complementary benchmark. It reflects a global cognitive evaluation of one’s quality of life [24], and it is widely used as a stable indicator of evaluative well-being. In Saudi Arabia, the Arabic version of the Satisfaction with Life Scale has demonstrated strong psychometric support [25]. Life satisfaction was not conceptualized as satisfaction with online experiences specifically; rather, it was treated as a domain-general indicator of evaluative well-being and as a distal convergent criterion. If perceived digital well-being captures a meaningful appraisal of how smartphone use supports emotional, social, and cognitive functioning in daily life, it should show a positive association with broader evaluations of life quality. Together, FoMO and life satisfaction enable a concise but informative assessment of convergent validity: perceived digital well-being should align negatively with FoMO and positively with life satisfaction.

### The present research

The present study aimed to psychometrically validate the Arabic version of the Perceived Digital Well-Being Scale (PDWS) among Saudi young adults aged (18–25). Specifically, the following objectives were pursued: (1) to examine the factor structure of the Arabic PDWS using exploratory and confirmatory factor analyses, including the second-order hierarchical specification; (2) to evaluate the reliability of domain scores and the total score using Cronbach’s alpha and McDonald’s omega; and (3) to assess convergent validity by examining the associations between PDWS scores and two theoretically relevant constructs—Fear of Missing Out (FoMO) and life satisfaction.

Based on the conceptual framework and prior validation evidence reviewed above, the following hypotheses were proposed: (H1) the Arabic PDWS will show a three-domain structure corresponding to Emotional, Social, and Cognitive perceived digital well-being, with these domains reflecting a broader higher-order perceived digital well-being construct; (H2) internal consistency reliability will be acceptable across domains and for the total score; and (H3) PDWS scores will show positive associations with life satisfaction and negative associations with FoMO, consistent with theoretical expectations.

## Materials and methods

### Study design and setting

The present study employed a cross-sectional instrument-validation design to examine the psychometric properties of the Arabic version of the Perceived Digital Well-Being Scale (PDWS) in Saudi young adults aged (18–25). Data were collected via an anonymous online survey administered in Riyadh, Saudi Arabia, between 15 March 2025 and 30 May 2025. Participants completed the PDWS along with two convergent validity measures: Fear of Missing Out (FoMO) and life satisfaction. A split-sample approach was used to provide a more rigorous cross-validation of the factor structure: exploratory factor analysis (EFA) was conducted in one subsample to identify the underlying structure, whereas confirmatory factor analysis (CFA) was conducted in an independent subsample to test the replicability and fit of the resulting measurement model. This approach reduces the risk of capitalizing on sample-specific variation when exploratory and confirmatory analyses are performed on the same participants.

### Participants

A total of (N = 450) Saudi young adults aged (18–25) participated in the study using a non-probabilistic convenience sampling approach, whereby participants were recruited through online dissemination of the survey link on social media platforms in Saudi Arabia. The full sample was then randomly split into two equal analytic subsamples: Sample 1 (EFA subsample; n = 225) and Sample 2 (CFA subsample; n = 225). The size of each subsample was considered adequate for the planned multivariate analyses. For EFA, Sample 1 provided approximately (13.2) participants per item for the (17)-item PDWS, exceeding the commonly used (10:1) participant-to-item heuristic. For CFA, Sample 2 included (225) cases and was considered sufficient for testing a (17)-item measurement model with three first-order domains and a second-order factor, given that CFA/SEM sample size requirements depend on model complexity, number of indicators, factor loadings, and related design features [26]. Exploratory factor analysis (EFA) was conducted in Sample 1, confirmatory factor analysis (CFA) was conducted in Sample 2, and convergent validity correlations were examined in both subsamples. Sociodemographic characteristics of Sample 1 (EFA subsample), Sample 2 (CFA subsample), and the total sample are presented in Table 1.

**Table 1:** Sociodemographic characteristics of the study sample (Sample 1, Sample 2, and total).

Characteristic	Sample 1 (EFA; n = 225)	Sample 2 (CFA; n = 225)	Total (N = 450)
Age (years)	(M = 22.27, SD = 1.87), range (18–25)	(M = 21.99, SD = 1.92), range (18–25)	(M = 22.13, SD = 1.90), range (18–25)
**Gender**			
Male	(115) (51.1%)	(111) (49.3%)	(226) (50.2%)
Female	(110) (48.9%)	(114) (50.7%)	(224) (49.8%)
**Marital status**			
Single	(161) (71.6%)	(157) (69.8%)	(318) (70.7%)
Married	(57) (25.3%)	(58) (25.8%)	(115) (25.6%)
Divorced/Widowed	(7) (3.1%)	(10) (4.4%)	(17) (3.8%)
**Education**			
High school or less	(40) (17.8%)	(39) (17.3%)	(79) (17.6%)
Bachelor	(152) (67.6%)	(148) (65.8%)	(300) (66.7%)
Postgraduate	(33) (14.7%)	(38) (16.9%)	(71) (15.8%)

***Note.*** *Values are (n) (%) unless otherwise stated. No missing data were observed for the reported sociodemographic variables in either subsample.*

In Sample 1 (EFA subsample), age ranged from (18) to (25) years (M = 22.27, SD = 1.87), with (115) males (51.1%) and (110) females (48.9%). Most participants were single (n = 161, 71.6%), followed by married (n = 57, 25.3%) and divorced/widowed (n = 7, 3.1%). Regarding education, (152) participants reported a bachelor’s degree (67.6%), (40) high school or less (17.8%), and (33) postgraduate education (14.7%).

In Sample 2 (CFA subsample), age ranged from (18) to (25) years (M = 21.99, SD = 1.92), with (111) males (49.3%) and (114) females (50.7%). Most participants were single (n = 157, 69.8%), followed by married (n = 58, 25.8%) and divorced/widowed (n = 10, 4.4%). Education levels included bachelor’s degree (n = 148, 65.8%), high school or less (n = 39, 17.3%), and postgraduate education (n = 38, 16.9%).

Across the full sample, the mean age was (M = 22.13, SD = 1.90), with (226) males (50.2%) and (224) females (49.8%). Overall marital status was (318) single (70.7%), (115) married (25.6%), and (17) divorced/widowed (3.8%). Education was distributed as (300) bachelor’s degree (66.7%), (79) high school or less (17.6%), and (71) postgraduate education (15.8%). No missing data were observed for the reported sociodemographic variables in either subsample.

### Ethical approval and consent

The study protocol was reviewed and approved by the Institutional Review Board (IRB), Imam Mohammad Ibn Saud Islamic University, Riyadh, Saudi Arabia (IRB registration: HAPO-01-R-061; Project No. 244/2025; approval date: 10 March 2025; full review). All participants provided electronic informed consent prior to participation. Participation was voluntary and responses were collected anonymously.

### Procedure

Participants were recruited through social media platforms, specifically WhatsApp, Snapchat, X (formerly Twitter), and Instagram, in Saudi Arabia between 15 March 2025 and 30 May 2025. Data were collected using a self-administered online questionnaire created and distributed via Google Forms. Participation was fully anonymous; no identifying information (e.g., names, phone numbers, email addresses, or student IDs) was collected. No incentives or compensation were provided. The survey required approximately (10–15) minutes to complete.

### Translation and cultural adaptation of the PDWS

The PDWS was translated and culturally adapted into Arabic using established cross-cultural test adaptation recommendations, with an emphasis on conceptual and semantic equivalence rather than literal translation [17,18]. First, two independent forward translations were produced by bilingual (Arabic–English) translators familiar with psychological and psychometric terminology. Second, a reconciliation meeting was conducted by the research team to compare the two Arabic versions item-by-item, resolve discrepancies, and agree on a single unified Arabic wording for each item, guided by clarity, accuracy of meaning, and suitability for Saudi young adults. Third, the unified Arabic version was reviewed by an expert panel (n = 10) specializing in psychology and psychometrics to evaluate linguistic clarity, cultural appropriateness, and alignment with the intended construct, resulting in minor wording refinements without changing the underlying meaning. Fourth, a blind back-translation into English was completed by an independent translator who had not accessed the original PDWS. The back-translated and original versions were compared to identify potential semantic deviations, and iterative adjustments were made to the Arabic items until satisfactory equivalence was achieved [27,28].

### Measures

#### Perceived digital well-being scale (PDWS).

Perceived digital well-being was assessed using the Perceived Digital Well-Being Scale (PDWS), a 17-item self-report measure originally comprising three domains: emotional digital well-being (7 items), social digital well-being (6 items), and cognitive digital well-being (4 items) [16]. Each item is presented in a bipolar format reflecting negative versus positive perceived outcomes of smartphone use, with responses recorded on a (5)-point scale ranging from (1) at the left pole to (5) at the right pole; higher scores indicate higher perceived digital well-being. Domain scores were computed by averaging the corresponding items within each domain, and a total PDWS score was computed by averaging all (17) items. In the present study, CFA in the independent CFA subsample supported a well-fitting second-order model (CFI = .986, RMSEA = .026, SRMR = .055), with all standardized factor loadings statistically significant (p < .001); full CFA results are reported in the Results section. Based on the EFA/CFA results, item (S11) was included in the Emotional domain when computing domain scores (Emotional = 8; Social = 5; Cognitive = 4). To illustrate the bipolar response format, a representative item is provided for each domain, anchored at 1 (negative pole) and 5 (positive pole): Emotional (E2)—“Because of my smartphone use, I feel more stressed” versus “Because of my smartphone use, I feel more relaxed”; Social (S8)—“Because of my smartphone use, I feel more excluded from my friends” versus “Because of my smartphone use, I feel closer to my friends”; Cognitive (C14)—“Because of my smartphone use, I do less of my daily tasks (e.g., schoolwork, university work, and professional work)” versus “Because of my smartphone use, I do more of my daily tasks (e.g., schoolwork, university work, and professional work).” The English version of the PDWS item set is provided in S1 Appendix.

### Convergent validity measures

#### Satisfaction with life scale (SWLS)

To assess participants’ global cognitive evaluation of life satisfaction, the Satisfaction with Life Scale (SWLS), originally developed by Diener et al. (1985), was used [24]. The present study employed the Arabic version validated for the Saudi Arabian context by Al-Dossary (2022) [25]. The SWLS is a (5)-item instrument rated on a (7)-point Likert-type scale ranging from (1 = Strongly Disagree) to (7 = Strongly Agree). Total scores range from (5–35), with higher scores indicating greater life satisfaction. In the Saudi context, Al-Dossary (2022) reported high internal consistency reliability (Cronbach’s alpha = .88 for students and .84 for employees, and McDonald’s omega values were .88 and .84, respectively). Evidence of validity was supported by a robust one-factor CFA solution with good fit, expected associations with well-being and happiness, negative correlations with depression indices, and established measurement invariance across gender, marital status, and role (students vs. employees) [25].

#### Fear of missing out scale (FoMO)

Fear of missing out was assessed using the FoMO Scale originally developed by Przybylski et al. (2013). This study employed an Arabic version supported by recent evidence from the Saudi context as reported by Al-Abyadh (2025) [23]. The FoMO Scale is a (10)-item self-report measure rated on a (5)-point Likert scale ranging from (1 = Not at all true of me) to (5 = Extremely true of me), with higher scores indicating higher levels of FoMO. Al-Abyadh (2025) reported satisfactory internal consistency in a Saudi sample and supported construct validity through theoretically consistent associations with related constructs, particularly social media addiction and psychological distress [23].

#### Confirmatory factor analysis model specification

Prior to CFA, item-level distributions in the CFA subsample were inspected to evaluate the suitability of maximum likelihood estimation for the five-point ordinal response format. All items had complete data (N = 225; no missing values). Skewness values ranged from (−0.860) to (0.078), and kurtosis values ranged from (−0.796) to (0.198), indicating no severe univariate non-normality. Given the five-point response format and the acceptable item-level distributional properties, maximum likelihood (ML) estimation was considered appropriate for the present CFA model; indeed, simulation evidence indicates that when ordinal items comprise at least five response categories and show approximately symmetric, near-normal distributions, treating them as continuous and estimating the model with ML yields parameter estimates, standard errors, and fit indices comparable to those obtained from categorical estimators such as diagonally weighted least squares (DWLS), whose relative advantages are most pronounced with fewer response categories and pronounced non-normality [29]. A second-order hierarchical model was specified rather than a bifactor model for three reasons. First, the PDWS was theoretically designed as a hierarchical construct in which domain-specific appraisals reflect an overarching perceived digital well-being factor [13,16]. Second, the observed and theoretically expected intercorrelations among the Emotional, Social, and Cognitive domains are more directly represented by a second-order structure, whereas a bifactor model would partition item variance into a general factor and residual specific factors. Third, second-order and bifactor models differ in the meaning of the general factor: in a second-order model, the general factor represents common variance shared through the first-order domains, whereas in a bifactor model, the general factor is estimated directly at the item level [30].

#### Measurement invariance

Measurement invariance across gender and education level was evaluated using multi-group CFA in a stepwise sequence: configural (same factor structure), metric (equal factor loadings), scalar (equal intercepts), and strict (equal residuals) invariance. Invariance was judged primarily using changes in fit indices between nested models (ΔCFI and ΔRMSEA), with thresholds of (ΔCFI≤0.010) and (ΔRMSEA≤0.015).

## Results

### Exploratory factor analysis (EFA) results

Exploratory factor analysis was conducted on the EFA subsample (n = 225) to examine the underlying factor structure of the Arabic PDWS. The data were suitable for factor analysis, as indicated by a high Kaiser–Meyer–Olkin (KMO) measure of sampling adequacy (.893) and a significant Bartlett’s test of sphericity (χ²(136)=1383.982, p < .001). The EFA was performed using maximum likelihood extraction with an oblique rotation (Direct Oblimin with Kaiser normalization). Factor retention was guided by the Kaiser-Guttman criterion (eigenvalues>1), visual inspection of the scree plot, and the maximum-likelihood goodness-of-fit test for the retained solution. The sampling adequacy and factorability indices for the EFA subsample are presented in Table 2.

**Table 2:** KMO and Bartlett’s Test of Sphericity (EFA Subsample, n = 225).

Test/Index	Value
Kaiser–Meyer–Olkin (KMO)	.893
Bartlett’s test of sphericity: χ²	1383.982
df	136
p	<.001

Both the Kaiser criterion (eigenvalues>1) and inspection of the scree plot supported a three-factor solution. The first three eigenvalues were (5.641), (2.440), and (1.175), respectively. Based on extraction sums of squared loadings, the three retained factors accounted for a cumulative (45.614%) of the extracted variance. The maximum likelihood goodness-of-fit test further supported the adequacy of the three-factor solution (χ²(88)=82.597, p = .643). Rotation converged in (6) iterations.

The rotated pattern matrix yielded a clear and interpretable structure with no salient cross-loadings, using a loading cutoff of (.30). Factor (1) consisted primarily of the emotional domain items (E1–E7), with loadings ranging from (.471–.689). Factor (2) consisted of the cognitive domain items (C14–C17), with strong loadings ranging from (.765–.838). Factor (3) consisted of the social domain items (S8–S10, S12–S13), with loadings ranging from (.320–.710). Item (S11) loaded on Factor (1) (loading = .715). The rotated pattern matrix, initial eigenvalues, and extracted variance for the three-factor solution are presented in Table 3.

**Table 3:** Rotated Pattern Matrix (Direct Oblimin) for the Arabic PDWS With Initial Eigenvalues and Extracted Variance (EFA Subsample, n = 225).

Item	Factor (1)	Factor (2)	Factor (3)
E1	.537	—	—
E2	.689	—	—
E3	.597	—	—
E4	.490	—	—
E5	.501	—	—
E6	.616	—	—
E7	.471	—	—
S8	—	—	.694
S9	—	—	.320
S10	—	—	.673
S11	.715	—	—
S12	—	—	.710
S13	—	—	.433
C14	—	.822	—
C15	—	.765	—
C16	—	.806	—
C17	—	.838	—
**Initial eigenvalue**	5.641	2.440	1.175
**% Variance extraction**	28.088	13.927	3.599

***Note*** *. Extraction method was maximum likelihood. Rotation method was Direct Oblimin with Kaiser normalization. Rotation converged in (6) iterations. “—” indicates coefficients below the reporting cutoff (.30). No salient cross-loadings were observed. The cumulative extracted variance explained by the three-factor solution was (45.614%).*

### Confirmatory factor analysis (CFA) results

Confirmatory factor analysis (CFA) was conducted on the CFA subsample (n = 225) using (AMOS 26) to verify the factor structure of the Arabic PDWS. The model was estimated using maximum likelihood (ML). Accordingly, a second-order model was specified in which Perceived Digital Well-Being was modeled as a higher-order construct reflected by three first-order dimensions (Emotional, Social, and Cognitive). The model demonstrated good fit to the data: χ²(116)=133.275, p = .130, with χ²/df=(1.149). Incremental fit indices indicated excellent fit (CFI = .986, TLI = .983, IFI = .986, NFI = .901), and residual-based fit was acceptable (SRMR = .055). The RMSEA also indicated excellent fit (RMSEA = .026, (90%) CI [.000, .044], PCLOSE = .990). The model fit indices for the second-order PDWS model are presented in Table 4.

**Table 4:** Confirmatory Factor Analysis Model Fit Indices for the Second-Order PDWS Model (CFA Subsample, n = 225).

Fit index	Value
χ² (df)	133.275 (116)
p	.130
χ²/df	1.149
CFI	.986
TLI	.983
IFI	.986
NFI	.901
SRMR	.055
RMSEA (90% CI)	.026 [.000, .044]
PCLOSE	.990

***Note*** *. The model was estimated using maximum likelihood (ML) in (AMOS 26). RMSEA confidence interval represents the (90%) CI reported by AMOS.*

All standardized factor loadings were statistically significant (p < .001). Standardized loadings ranged from (.422–.683) for the Emotional dimension, (.574–.731) for the Social dimension, and (.738–.791) for the Cognitive dimension. Consistent with the EFA results, item (S11) loaded on the Emotional factor (standardized loading = .603, p < .001). Squared multiple correlations (R²) indicated that the indicators explained meaningful proportions of variance, with R² values ranging from (.178–.467) for Emotional items, (.330–.534) for Social items, and (.545–.626) for Cognitive items. The standardized item loadings and squared multiple correlations are presented in Table 5.

**Table 5:** Standardized Factor Loadings and Squared Multiple Correlations (R²) for PDWS Items (Second-Order CFA; CFA Subsample, n = 225).

First-order factor	Item	Standardized loading	R² (SMC)
Emotional	E1	.498	.248
	E2	.683	.467
	E3	.620	.384
	E4	.466	.217
	E5	.652	.426
	E6	.567	.321
	E7	.422	.178
	S11	.603	.364
Social	S8	.724	.525
	S9	.574	.330
	S10	.671	.450
	S12	.621	.386
	S13	.731	.534
Cognitive	C14	.738	.545
	C15	.783	.613
	C16	.791	.626
	C17	.766	.586

***Note*** *. All standardized loadings were statistically significant (p < .001). R² values are squared multiple correlations (SMC) reported by AMOS.*

At the second-order level, the higher-order PDWS factor showed strong standardized loadings on the three first-order dimensions (Emotional = .894, Social = .749, Cognitive = .759), all statistically significant (p < .001), supporting the interpretation of PDWS as a higher-order construct reflected by the three domains. The standardized second-order loadings are presented in Table 6.

**Table 6:** Standardized Second-Order Loadings of PDWS on the First-Order Dimensions (CFA Subsample, n = 225).

Higher-order factor	First-order dimension	Standardized loading	R² (SMC)
Perceived Digital Well-Being	Emotional	.894	.799
	Social	.749	.561
	Cognitive	.759	.576

***Note*** *. Second-order loadings are standardized estimates from the second-order CFA model. All second-order loadings were statistically significant (p < .001). R² values are squared multiple correlations reported by AMOS.*

**Fig 1:**
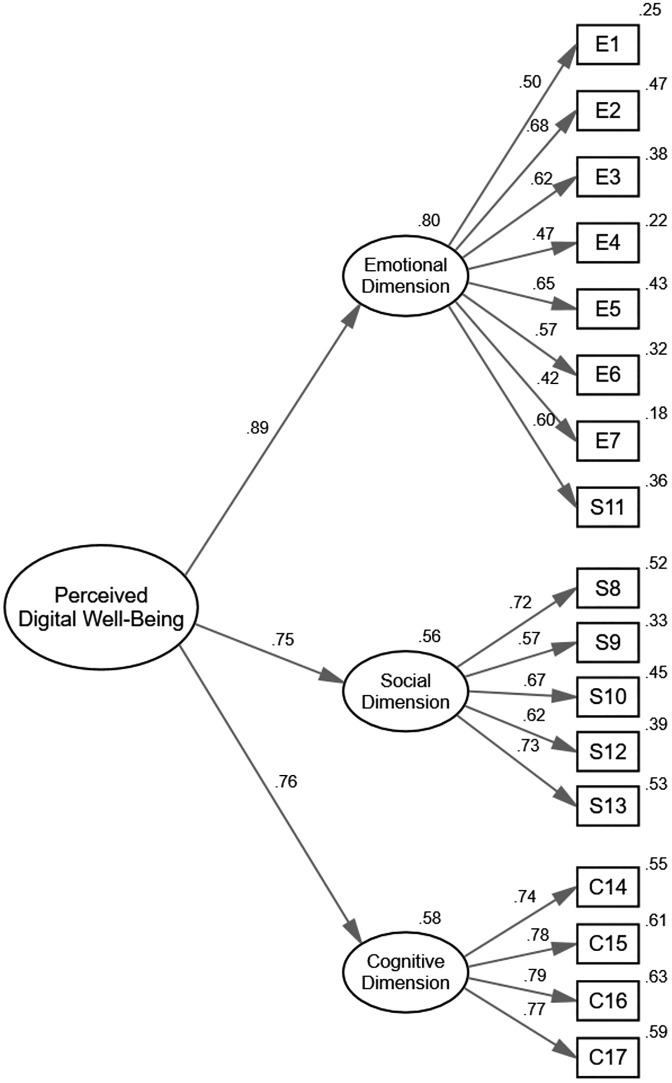
Standardized second-order CFA model of the Arabic PDWS (n = 225). Standardized loadings are shown on paths; values above items are R^2^.

The standardized path diagram for the second-order CFA model is shown in Fig 1.

### Convergent validity (SWLS and FoMO)

Convergent validity of the Arabic PDWS was examined by correlating PDWS scores with (SWLS) and (FoMO) separately in Sample 1 (EFA subsample; n = 225) and Sample 2 (CFA subsample; n = 225). In Sample 1, PDWS domain scores and the total PDWS score were positively associated with life satisfaction (SWLS) (r range = .296–.432, p ≤ .01) and negatively associated with fear of missing out (FoMO) (r range = −.141 to −.208, p ≤ .05). In Sample 2, the same pattern was replicated with larger magnitudes: PDWS scores correlated positively with SWLS (r range = .416–.550, p < .01) and negatively with FoMO (r range = −.254 to −.321, p < .01). Overall, these findings support convergent validity by demonstrating theoretically consistent associations across two independent subsamples, as shown in Table 7.

**Table 7:** Convergent Validity Correlations Between PDWS Scores, SWLS, and FoMO Across Two Independent Subsamples.

PDWS	Sample 1 (EFA; n = 225)		Sample 2 (CFA; n = 225)	
	SWLS	FoMO	SWLS	FoMO
Emotional	.353**	−.141*	.453**	−.254**
Social	.361**	−.189**	.416**	−.269**
Cognitive	.296**	−.162*	.495**	−.271**
PDWS	.432**	−.208**	.550**	−.321**

***Note*** . Pearson correlations are reported. * p < .05. ** p < .01 (two-tailed)

### Reliability

Internal consistency reliability of the Arabic PDWS was evaluated in both subsamples using Cronbach’s alpha (α) and McDonald’s omega (ω). In Sample 1 (EFA subsample; n = 225), reliability estimates were acceptable to excellent across the three dimensions (α range = .759–.885; ω range = .761–.889), and for the total PDWS score (α = .872; ω = .861). In Sample 2 (CFA subsample; n = 225), internal consistency was similarly acceptable across dimensions (α range = .788–.850; ω range = .787–.851) and was strong for the total score (α = .886; ω = .883). Overall, these results indicate that the Arabic PDWS yields reliable scores at both the domain and total-score levels across two independent subsamples, as shown in Table 8.

**Table 8:** Internal Consistency Reliability (Cronbach’s α and McDonald’s ω) for the PDWS Across Two Independent Subsamples.

PDWS	Items (k)	Sample 1 (EFA; n = 225)		Sample 2 (CFA; n = 225)	
		Cronbach’s α	McDonald’s ω	Cronbach’s α	McDonald’s ω
Emotional	(8)	.822	.822	.788	.787
Social	(5)	.759	.761	.797	.799
Cognitive	(4)	.885	.889	.850	.851
PDWS	(17)	.872	.861	.886	.883

***Note*** *. Cronbach’s α and McDonald’s ω are reported for each dimension and for the total PDWS score.*

### Construct reliability and validity

Construct-level reliability and validity of the Arabic PDWS were evaluated in the CFA subsample (Sample 2; n = 225) using composite reliability (CR), average variance extracted (AVE), and the Fornell–Larcker criterion. As shown in Table 9, CR values indicated good construct reliability across the three dimensions (CR range = .790–.853). AVE values, however, were heterogeneous: the Cognitive dimension exceeded the conventional .50 threshold (AVE = .593), whereas the Emotional (AVE = .326) and Social (AVE = .445) dimensions fell below it. Consequently, the Fornell–Larcker criterion was only partially met. The square root of the AVE for the Cognitive dimension (.770) exceeded its latent correlations with the Emotional (.678) and Social (.568) dimensions, supporting its discriminant distinctiveness. By contrast, the square root of the AVE for the Emotional dimension (.571) was lower than its correlations with the Social (.669) and Cognitive (.678) dimensions, and the square root of the AVE for the Social dimension (.667) was marginally lower than its correlation with the Emotional dimension (.669). Thus, the Fornell–Larcker discriminant validity criterion was not satisfied for the Emotional and Social dimensions, indicating that at the first-order level these two domains shared more variance with one another than each captured from its own indicators [31].

**Table 9:** Construct Reliability and Validity (Sample 2; n = 225).

Construct	CR	AVE	(1) Emotional	(2) Social	(3) Cognitive
**(1) Emotional**	.790	.326	**.571**		
**(2) Social**	.799	.445	.669	**.667**	
**(3) Cognitive**	.853	.593	.678	.568	**.770**

***Note*** *. CR = composite reliability; AVE = average variance extracted; Diagonal values are √AVE; off-diagonal values are standardized latent factor correlations from AMOS.*

### Measurement invariance across gender and education

Measurement invariance analyses supported the equivalence of the (PDWS) measurement model across gender and education level. Across gender, fit indices remained stable from the configural to strict models (Table 10), with small changes in fit (maximum (ΔCFI = 0.007) and (ΔRMSEA = 0.002)), supporting scalar and strict invariance. Across education level (three groups), invariance was also supported (Table 11). The metric step showed a borderline but acceptable change (ΔCFI = 0.010; ΔRMSEA = 0.001), and subsequent steps remained within recommended thresholds, supporting scalar and strict invariance across education level. Together, these findings indicate that the (PDWS) operates comparably across males and females and across education groups, supporting meaningful group comparisons.

**Table 10:** Measurement invariance of the (PDWS) across gender (Male vs Female).

Model	χ²(df)	CFI	RMSEA	ΔCFI	ΔRMSEA
Configural (Unconstrained)	289.495(232)	0.954	0.033	—	—
Metric (Measurement weights)	302.934(246)	0.954	0.032	0.000	0.001
Scalar (Measurement intercepts)	329.165(263)	0.947	0.034	0.007	0.002
Strict (Measurement residuals)	354.233(286)	0.945	0.033	0.002	0.001

***Note.*** *Δ values are absolute changes relative to the previous model.*

**Table 11:** Measurement invariance of the (PDWS) across education level (High school or less vs Bachelor vs Postgraduate).

Model	χ²(df)	CFI	RMSEA	ΔCFI	ΔRMSEA
Configural (Unconstrained)	426.552(348)	0.938	0.032	—	—
Metric (Measurement weights)	466.526(376)	0.928	0.033	0.010	0.001
Scalar (Measurement intercepts)	491.384(410)	0.936	0.030	0.008	0.003
Strict (Measurement residuals)	533.296(456)	0.939	0.028	0.003	0.002

***Note.*** *Δ values are absolute changes relative to the previous model.*

## Discussion

The present study provides initial evidence that the Arabic version of the Perceived Digital Well-Being Scale (PDWS) is a psychometrically sound instrument for assessing Saudi young adults’ perceived smartphone-related well-being. Using a split-sample design, the exploratory results supported a clear three-factor structure corresponding to the Emotional, Social, and Cognitive dimensions, and the confirmatory results further supported a hierarchical conceptualization in which these three domains are explained by an overarching perceived digital well-being factor. This pattern is consistent with the scale’s theoretical premise that individuals form domain-specific appraisals of smartphone use (e.g., emotions, relationships, and day-to-day functioning) that cohere into a broader subjective evaluation of digital well-being [13,16].

From a structural standpoint, sampling adequacy and factorability indicators were strong, supporting the suitability of the item set for latent structure examination. The EFA results aligned with the intended three-domain solution, and item loadings exceeded the adopted cutoff, with no evidence of problematic cross-loadings. The CFA results provided further support for the measurement model, with fit indices indicating excellent global fit (e.g., CFI = .986, RMSEA = .026, SRMR = .055, and CMIN/DF = 1.149), consistent with commonly cited criteria for well-fitting CFA models [32]. Importantly, the second-order specification was empirically justified by the strong loadings of the three first-order factors on the general PDWS factor, supporting the use of both domain scores and a total PDWS score when the research aim concerns overall perceived digital well-being.

Item-level saturation, as indexed by squared multiple correlations (R^2^/SMC) in Table 5, also provides insight into how strongly individual items were explained by their latent domains. Cognitive items showed the strongest and most consistent explained variance (R^2^ = .545–.626), suggesting that perceived cognitive consequences of smartphone use formed a relatively coherent component of digital well-being in this sample. Social items showed moderate-to-strong values (R^2^ = .330–.534), whereas Emotional items were more variable (R^2^ = .178–.467). This pattern suggests that emotional appraisals of smartphone use may be more heterogeneous and context-sensitive than cognitive appraisals, which is plausible given that digital engagement can elicit diverse affective responses such as reassurance, stress, enjoyment, comparison, or distress. Thus, the item-level results support the view that the Arabic PDWS captures a multidimensional appraisal of smartphone-related functioning rather than a single undifferentiated experience of digital well-being.

A notable finding was that the item reflecting the perceived impact of smartphone use on “true friendships” (S11) aligned more strongly with the Emotional dimension in the Saudi sample, rather than the Social dimension as originally proposed. Conceptually, this pattern is defensible because appraisals about the authenticity of friendships may function primarily as affect-laden evaluations (e.g., feelings of reassurance, insecurity, comfort, or emotional safety) rather than as a purely interactional indicator of social contact frequency or connection. In relationally oriented settings, where the quality of social bonds is central to emotional stability and day-to-day affect, reflections on “true friendships” may be experienced as an emotional consequence of smartphone use (e.g., worry, calmness, satisfaction, or distress), thereby clustering with other affective outcomes. Consistent with this item-level behavior, domain scoring and reliability estimation in the present study reflected the empirically supported allocation of S11 to the Emotional domain. This culturally plausible interpretation does not undermine the model; instead, it illustrates the value of cross-cultural validation in testing whether item–factor alignment remains stable across contexts, as recommended in test adaptation guidance [18].

Convergent validity evidence was also consistent with theory. PDWS scores—both at the domain and total levels—showed positive associations with life satisfaction as measured by the SWLS, indicating that more favorable perceived smartphone-related well-being is linked to higher global cognitive evaluations of one’s life [24,25]. At the same time, PDWS scores were negatively associated with FoMO, suggesting that individuals who experience greater fear of missing out tend to report poorer perceived digital well-being, in line with FoMO models emphasizing persistent social-monitoring concerns and their psychological costs [21,23]. The replication of these associations across both subsamples strengthens confidence that the nomological network of the Arabic PDWS aligns with theoretically relevant constructs.

It is also important to interpret the magnitude of the convergent validity correlations. Although all associations were statistically significant and directionally consistent across both independent subsamples, most were small to moderate in magnitude (Sample 1: |r| = .141–.432; Sample 2: |r| = .254–.550; Table 7). This pattern is theoretically reasonable because SWLS and FoMO were used as distal convergent criteria rather than measures of the same construct. SWLS reflects a broad evaluation of life quality, whereas FoMO reflects a specific social-monitoring tendency; both are expected to relate to perceived digital well-being without being redundant with it. Moreover, the broader digital technology and well-being literature has consistently shown that associations between digital engagement and psychological outcomes are often small on average [9–12]. Therefore, the primary validity evidence lies in the replicated direction of the associations across two independent subsamples—positive with life satisfaction and negative with FoMO—rather than in expecting uniformly strong correlations. These weak-to-moderate magnitudes suggest that the Arabic PDWS captures a related but distinct appraisal of smartphone-related functioning.

Regarding reliability, internal consistency estimates were acceptable to strong for the PDWS domains and the total score across both subsamples, with Cronbach’s alpha and McDonald’s omega largely converging—an encouraging pattern given that omega is often preferred when congeneric measurement is expected. In addition, construct reliability indicators derived from the CFA model (CR) supported adequate construct-level reliability. AVE values were heterogeneous across domains—below the conventional .50 threshold for the Emotional (.326) and Social (.445) dimensions and above it for the Cognitive dimension (.593)—and, consequently, the Fornell–Larcker discriminant validity criterion was not fully satisfied. Specifically, the square root of the AVE for the Emotional dimension (.571) was exceeded by its latent correlations with the Social (.669) and Cognitive (.678) dimensions, and the square root of the AVE for the Social dimension (.667) was marginally exceeded by its correlation with the Emotional dimension (.669). This pattern indicates that, at the first-order level, the Emotional and Social domains shared more variance with each other than each explained in its own indicators. Rather than signaling a structural flaw, this outcome is theoretically coherent with the second-order specification adopted in the present study: when first-order domains function as strong reflective indicators of an overarching factor, their inter-domain correlations are expected to be high, which mechanically constrains first-order discriminant validity as indexed by the Fornell–Larcker criterion. Consistent with this interpretation, the three domains loaded strongly on the higher-order PDWS factor (Emotional = .894, Social = .749, Cognitive = .759; Table 6), indicating substantial common variance shared through the general construct. Accordingly, the elevated inter-domain correlations are more appropriately interpreted as evidence for the hierarchical structure of perceived digital well-being than as a failure of the measurement model, although the modest AVE of the Emotional and Social domains should be interpreted with caution [31,33]. In this regard, presenting both internal consistency (alpha/omega) and model-based reliability/validity indices (CR/AVE) provides a transparent evaluation of measurement quality, consistent with contemporary psychometric reporting expectations in scale validation work [19].

Overall, the findings support the Arabic PDWS as a practical tool for assessing perceived smartphone-related well-being among Saudi young adults, offering researchers and practitioners a balanced measurement approach that moves beyond a sole focus on problematic use and toward understanding how individuals perceive the benefits and costs of smartphone engagement across emotional, social, and cognitive life domains [13,16,34].

### Practical implications

The validation of the Arabic PDWS has several practical implications for the Saudi context. First, it provides researchers with a culturally adapted Arabic instrument for assessing perceived smartphone-related digital well-being among Saudi young adults. This is particularly useful in a rapidly digitalizing society where smartphone use is closely embedded in learning, communication, peer relationships, and daily routines. By assessing perceived benefits and costs across emotional, social, and cognitive domains, the PDWS offers a more balanced alternative to measures that focus only on problematic or excessive use.

Second, the Arabic PDWS may be useful in university counseling, student well-being services, and psychoeducational programs. Rather than identifying digital difficulties only through screen time or generalized problematic-use indicators, the scale can help clarify whether perceived digital strain is primarily emotional, social, or cognitive. This domain-level information may guide more targeted interventions, such as programs on digital self-regulation, healthy responsiveness norms, managing FoMO, boundary-setting in online relationships, and maintaining attention and productivity during academic tasks.

Third, at the institutional and policy level, the Arabic PDWS may support evidence-based monitoring of perceived digital well-being among Saudi young adults. The scale could be used to evaluate digital literacy and well-being initiatives, compare patterns across student groups, and identify subgroups who may benefit from preventive support. In this way, the Arabic PDWS can contribute to research, counseling practice, and institutional planning aimed at promoting healthier digital engagement while preserving the educational and social benefits of connectivity in the Saudi context.

### Limitations and future directions

Several limitations should be acknowledged. First, participants were recruited online through social media and completed the survey anonymously via Google Forms. Although this approach enabled efficient access to the target age group, it may have introduced self-selection bias and limits the representativeness of the sample. Second, the study was conducted in Riyadh and focused on Saudi young adults aged (18–25); therefore, generalization to other regions, age groups, or Arab populations should be made cautiously. Third, the cross-sectional design precludes causal conclusions regarding the observed associations between perceived digital well-being, FoMO, and life satisfaction. Fourth, reliability evidence in the present study was limited to internal consistency; future work should examine temporal stability using test–retest designs. In addition, although construct reliability was adequate, the average variance extracted for the Emotional and Social domains fell below the conventional threshold and the first-order Fornell–Larcker discriminant validity criterion was not fully met; future studies should further examine first-order discriminant validity using complementary discriminant-validity indices and larger, more diverse samples. Future studies could also apply item response theory (IRT) models, such as the graded response model, or network psychometric approaches to examine item-level discrimination, threshold parameters, response category functioning, and item-to-item relationships, thereby complementing the present SEM-based evidence from CFA and measurement invariance analyses. Finally, measurement invariance across gender and education level was supported in the present study; however, additional validity evidence remains warranted, including predictive validity in relation to theoretically relevant outcomes that capture both adaptive and strain-related aspects of digital experience (e.g., sleep quality, academic/work performance, psychological distress, and digital stress; [35]) and replication in broader Saudi and Arabic-speaking samples. The placement of item (S11) on the Emotional domain also highlights the importance of continued evaluation of item functioning across contexts, including replication in broader Saudi samples and across other Arabic-speaking populations.

## Conclusion

This study provides initial psychometric support for the Arabic version of the Perceived Digital Well-Being Scale (PDWS) among Saudi young adults aged (18–25). Using a split-sample approach, the findings supported a clear three-domain structure and a well-fitting second-order model, indicating that emotional, social, and cognitive appraisals of smartphone use coherently reflect an overarching perceived digital well-being construct. The Arabic PDWS demonstrated theoretically consistent convergent validity through positive associations with life satisfaction (SWLS) and negative associations with Fear of Missing Out (FoMO), and it showed acceptable internal consistency across domains and the total score in two independent subsamples. Future research should extend this evidence by examining temporal stability and predictive validity for relevant outcomes in Saudi and other Arabic-speaking contexts.

### Artificial intelligence statement

During manuscript revision, ChatGPT (OpenAI) was used to support language editing, organization of reviewer responses, and refinement of selected manuscript text under the author’s supervision. The tool was not used to generate data, conduct statistical analyses, create or alter scientific figures, or determine the study findings. The author reviewed, verified, and approved all AI-assisted text and remains fully responsible for the accuracy, integrity, citations, analyses, and conclusions of the manuscript.

## Supporting information

S1 Appendix Perceived Digital Well-Being Scale (PDWS): English version (CC BY 4.0). (PDF)
