# Supplementary material for: Psychometric properties of the Arabic Perceived Digital Well-Being Scale (PDWS) in Saudi young adults
Source: PLoS One. 2026 Jul 30;21(7):e0354474. doi: 10.1371/journal.pone.0354474 (PMC13422880; doi:10.1371/journal.pone.0354474)
Supplement: S1 Appendix — (PDF) [file pone.0354474.s001.pdf]

S1 Appendix

Perceived Digital Well-Being Scale (PDWS): English version

(CC BY 4.0)

Source and license

This appendix presents the English version of the Perceived Digital Well-Being Scale (PDWS). The item wording is reproduced from the open-access psychometric validation study by Vera Cruz et al. (2025), which is distributed under the Creative Commons Attribution License (CC BY 4.0).

Source: Vera Cruz G, Liberacka-Dwojak M, Wiłkość-Dębczyńska M, Aktaş Terzioğlu M, Farchione T, Lecomte T, Ingram S, Khan R, Khazaal Y. Perceived Digital Well-Being Scale in the United States and United Kingdom: Psychometric Validation Study. JMIR Mental Health. 2025;12:e78334. doi: 10.2196/78334.

The scale consists of 17 bipolar self-report items covering three domains: emotional digital well-being, social digital well-being, and cognitive digital well-being. Responses are recorded on a 5-point scale from 1 (left pole) to 5 (right pole). Higher scores indicate higher perceived digital well-being.

Scale items

Domain	Item	Item wording
Emotional domain (ED)	E1	Because of my smartphone use, I do fewer fun things, or because of my smartphone use, I do more fun things.
Emotional domain (ED)	E2	Because of my smartphone use, I feel more stressed, or because of my smartphone use, I feel more relaxed.
Emotional domain (ED)	E3	Because of my smartphone use, I feel bored, or because of my smartphone use, I feel entertained.
Emotional domain (ED)	E4	Because of my smartphone use, I drop some leisure activities that I like, or because of my smartphone use, I engage in new leisure activities that I like.
Emotional domain (ED)	E5	Because of my smartphone use, I feel more upset, or because of my smartphone use, I feel calmer.
Emotional domain (ED)	E6	Because of my smartphone use, I feel sadder, or because of my smartphone use, I feel happier.
Emotional domain (ED)	E7	Because of my smartphone use, I feel my life is worse than most teens, or because of my smartphone use, I feel my life is better than most teens.
Social domain (SD)	S8	Because of my smartphone use, I feel more excluded from my friends, or because of my smartphone use, I feel closer to my friends.
Social domain (SD)	S9	Because of my smartphone use, I talk less with my friends, or because of my smartphone use, I talk more with my friends.
Social domain (SD)	S10	Because of my smartphone use, I feel less informed about the lives of my friends, or because of my smartphone use, I feel more informed about the lives of my friends.
Social domain (SD)	S11	Because of my smartphone use, I have fewer true friendships, or because of my smartphone use, I have more true friendships.

Social domain (SD)	S12	Because of my smartphone use, I feel less connected to my friends, or because of my smartphone use, I feel more connected to my friends.
Social domain (SD)	S13	Because of my smartphone use, I spend less time with my friends, or because of my smartphone use, I spend more time with my friends.
Cognitive domain (CD)	C14	Because of my smartphone use, I do less of my daily tasks (eg, schoolwork, university work, and professional work), or because of my smartphone use, I do more of my daily tasks (eg, schoolwork, university work, and professional work).
Cognitive domain (CD)	C15	Because of my smartphone use, I have lower grades at school, at university, or low performance when doing important work, or because of my smartphone use, I have high grades at school, at university, or high performance when doing important work.
Cognitive domain (CD)	C16	Because of my smartphone use, I am slower in my daily tasks (eg, schoolwork, university work, and professional work), or because of my smartphone use, I am faster in my daily tasks (eg, schoolwork, university work, and professional work).
Cognitive domain (CD)	C17	Because of my smartphone use, I feel I am a failure, or because of my smartphone use, I feel satisfied with myself.

Scoring note for the present Arabic validation study

In the original English PDWS item listing, items E1-E7 represent the emotional domain, items S8-S13 represent the social domain, and items C14-C17 represent the cognitive domain. In the present Arabic validation study, item S11 loaded on the Emotional factor and was therefore included in the Emotional domain when computing domain scores, as reported in the manuscript.

This appendix is provided as supporting information for the manuscript: Psychometric properties of the Arabic Perceived Digital Well-Being Scale (PDWS) in Saudi young adults.
