# Supplementary material for: Psychometric properties of the Arabic Perceived Digital Well-Being Scale (PDWS) in Saudi young adults
Source: PLoS One. 2026 Jul 30;21(7):e0354474. doi: 10.1371/journal.pone.0354474 (PMC13422880; doi:10.1371/journal.pone.0354474)
Supplement: Attachment — Submitted filename: PONE-D-26-11267_reviewer.pdf [file pone.0354474.s003.pdf]

PLOS One

Psychometric Properties of the Arabic Perceived Digital Well-Being Scale (PDWS) in Saudi Young Adults

--Manuscript Draft--

**Manuscript Number:**	PONE-D-26-11267
**Article Type:**	Research Article
**Full Title:**	Psychometric Properties of the Arabic Perceived Digital Well-Being Scale (PDWS) in Saudi Young Adults
**Short Title:**	Arabic PDWS Psychometric Properties in Saudi Young Adults
**Corresponding Author:**	Emadeldin M. Elsokkary, Ph.D<br>Imam Mohammad Ibn Saud Islamic University (IMSIU)<br>Riyadh, SAUDI ARABIA
**Keywords:**	Perceived Digital Well-Being; Scale Validation; Saudi Young Adults; Fear of Missing Out; life satisfaction
**Abstract:**	Rapid digital transformation in Saudi Arabia has intensified young adults' day-to-day reliance on smartphones, increasing the need for balanced, culturally appropriate measures of perceived digital well-being. This study aimed to psychometrically validate the Arabic version of the Perceived Digital Well-Being Scale (PDWS) among Saudi young adults aged (18–25). A cross-sectional instrument-validation study was conducted with (N=450) participants who were randomly split into two independent subsamples: Sample (1) for exploratory factor analysis (EFA) (n=225) and Sample (2) for confirmatory factor analysis (CFA) and convergent validity testing (n=225). EFA supported a clear three-factor structure (Emotional, Social, Cognitive). CFA confirmed a second-order model with excellent fit: $\chi2(116)=133.275$, p=.130, $\chi2/df=(1.149)$, CFI=(.986), TLI=(.983), RMSEA=(.026), and SRMR=(.055). Convergent validity was supported by positive associations with life satisfaction (SWLS) and negative associations with fear of missing out (FoMO), with the same pattern observed across both subsamples (Sample (2): SWLS r=(.416–.550), p<.01; FoMO r=(-.321–-.254), p<.01). Internal consistency was acceptable across subsamples (Cronbach's α=(.759–.886); McDonald's ω=(.761–.889)). Overall, the Arabic PDWS demonstrates robust structural validity, theoretically consistent convergent validity, and adequate reliability, supporting its use as a multidimensional measure of perceived smartphone-related digital well-being among Saudi emerging adults.
**Order of Authors:**	Emadeldin M. Elsokkary, Ph.D
**Additional Information:**	
**Question**	**Response**
**Financial Disclosure**<br><br>Enter a financial disclosure statement that describes the sources of funding for the work included in this submission. Review the submission guidelines for detailed requirements. View published research articles from *PLOS ONE* for specific examples.<br><br>This statement is required for submission and **will appear in the published article** if the submission is accepted. Please make sure it is accurate.	Yes

Powered by Editorial Manager® and ProduXion Manager® from Aries Systems Corporation

**Funded studies**<br>Enter a statement with the following details:<br>• Initials of the authors who received each award<br>• Grant numbers awarded to each author<br>• The full name of each funder<br>• URL of each funder website<br>• Did the sponsors or funders play any role in the study design, data collection and analysis, decision to publish, or preparation of the manuscript?<br><br>Did you receive funding for this work?	
Please add funding details.<br>as follow-up to "**Financial Disclosure**<br><br>Enter a financial disclosure statement that describes the sources of funding for the work included in this submission. Review the submission guidelines for detailed requirements. View published research articles from *PLOS ONE* for specific examples.<br><br>This statement is required for submission and **will appear in the published article** if the submission is accepted. Please make sure it is accurate.<br><br>**Funded studies**<br>Enter a statement with the following details:<br>• Initials of the authors who received each award<br>• Grant numbers awarded to each author<br>• The full name of each funder<br>• URL of each funder website<br>• Did the sponsors or funders play any role in the study design, data collection and analysis, decision to publish, or preparation of the manuscript?<br><br>Did you receive funding for this work?"	This work was supported and funded by the Deanship of Scientific Research at Imam Mohammad Ibn Saud Islamic University (IMSIU) (grant number IMSIU-DDRSP2601).
Please select the country of your main research funder (please select carefully as in some cases this is used in fee calculation).	SAUDI ARABIA - SA

as follow-up to "**Financial Disclosure**<br><br>Enter a financial disclosure statement that describes the sources of funding for the work included in this submission. Review the submission guidelines for detailed requirements. View published research articles from *PLOS ONE* for specific examples.<br><br>This statement is required for submission and **will appear in the published article** if the submission is accepted. Please make sure it is accurate.<br><br>**Funded studies**<br>Enter a statement with the following details:<br>• Initials of the authors who received each award<br>• Grant numbers awarded to each author<br>• The full name of each funder<br>• URL of each funder website<br>• Did the sponsors or funders play any role in the study design, data collection and analysis, decision to publish, or preparation of the manuscript?<br><br>Did you receive funding for this work?"	
**Competing Interests**<br><br>Use the instructions below to enter a competing interest statement for this submission. On behalf of all authors, disclose any competing interests that could be perceived to bias this work—acknowledging all financial support and any other relevant financial or non-financial competing interests.<br><br>This statement is required for submission and **will appear in the published article** if the submission is accepted. Please make sure it is accurate and that any funding sources listed in your Funding Information later in the submission form are also declared in your Financial Disclosure statement.	The authors have declared that no competing interests exist.

View published research articles from [PLOS ONE](#) for specific examples.<br><br>**NO authors have competing interests**<br><br>Enter: *The authors have declared that no competing interests exist.*<br><br>**Authors with competing interests**<br><br>Enter competing interest details beginning with this statement:<br><br>*I have read the journal's policy and the authors of this manuscript have the following competing interests: [insert competing interests here]*<br><br>* typeset	
**Ethics Statement**<br><br>Enter an ethics statement for this submission. This statement is required if the study involved:<br><br>• Human participants<br>• Human specimens or tissue<br>• Vertebrate animals or cephalopods<br>• Vertebrate embryos or tissues<br>• Field research<br><br>Write "N/A" if the submission does not require an ethics statement.<br><br>General guidance is provided below. Consult the submission guidelines for detailed instructions. **Make sure that all information entered here is included in the Methods section of the manuscript.**	The study protocol was reviewed and approved by the Institutional Review Board (IRB), Al-Imam Muhammad ibn Saud Islamic University, Riyadh, Saudi Arabia (IRB registration: HAPO-01-R-061; Project No. 244/2025; approval date: 10 March 2025; full review).<br>All participants (Saudi young adults aged 18–25 years) provided informed consent prior to participation. Participation was voluntary and responses were collected anonymously.

**Format for specific study types**<br><br>**Human Subject Research (involving human participants and/or tissue)**<br>• Give the name of the institutional review board or ethics committee that approved the study<br>• Include the approval number and/or a statement indicating approval of this research<br>• Indicate the form of consent obtained (written/oral) or the reason that consent was not obtained (e.g. the data were analyzed anonymously)<br><br>**Animal Research (involving vertebrate animals, embryos or tissues)**<br>• Provide the name of the Institutional Animal Care and Use Committee (IACUC) or other relevant ethics board that reviewed the study protocol, and indicate whether they approved this research or granted a formal waiver of ethical approval<br>• Include an approval number if one was obtained<br>• If the study involved *non-human primates*, add *additional details* about animal welfare and steps taken to ameliorate suffering<br>• If anesthesia, euthanasia, or any kind of animal sacrifice is part of the study, include briefly which substances and/or methods were applied<br><br>**Field Research**<br><br>Include the following details if this study involves the collection of plant, animal, or other materials from a natural setting:<br>• Field permit number<br>• Name of the institution or relevant body that granted permission	
**Data Availability**<br><br>Authors are required to make all data underlying the findings described fully available, without restriction, and from the time of publication. PLOS allows rare exceptions to address legal and ethical concerns. See the PLOS Data Policy and FAQ for detailed information.	Yes - all data are fully available without restriction

Powered by Editorial Manager® and ProduXion Manager® from Aries Systems Corporation

A Data Availability Statement describing where the data can be found is required at submission. Your answers to this question constitute the Data Availability Statement and **will be published in the article**, if accepted.<br><br>**Important:** Stating 'data available on request from the author' is not sufficient. If your data are only available upon request, select 'No' for the first question and explain your exceptional situation in the text box.<br><br>Do the authors confirm that all data underlying the findings described in their manuscript are fully available without restriction?	
**Describe where the data may be found in full sentences. If you are copying our sample text, replace any instances of XXX with the appropriate details.**<br><br>• If the data are **held or will be held in a public repository**, include URLs, accession numbers or DOIs. If this information will only be available after acceptance, indicate this by ticking the box below. For example: *All XXX files are available from the XXX database (accession number(s) XXX, XXX.).*<br>• If the data are all contained **within the manuscript and/or Supporting Information files**, enter the following: *All relevant data are within the manuscript and its Supporting Information files.*<br>• If neither of these applies but you are able to provide **details of access elsewhere**, with or without limitations, please do so. For example:<br><br>*Data cannot be shared publicly because of [XXX]. Data are available from the XXX Institutional Data Access / Ethics Committee (contact via XXX) for researchers who meet the criteria for access to confidential data.*<br><br>*The data underlying the results presented in the study are available from (include the name of the third party*	All data underlying the findings described in this manuscript are publicly available in Zenodo (doi: 10.5281/zenodo.18882971).

and contact information or URL).<br>• This text is appropriate if the data are owned by a third party and authors do not have permission to share the data.<br><br>* typeset	
Additional data availability information:	

Powered by Editorial Manager® and ProduXion Manager® from Aries Systems Corporation

Manuscript Click here to access/download;Manuscript;Manuscript 18-3-2026.docx

Psychometric Properties of the Arabic Perceived Digital Well-Being Scale (PDWS) in Saudi Young Adults

Emadeldin M. Elsokkary[(1)]

[(1)] Department of Psychology, College of Social Sciences, Imam Mohammad Ibn Saud Islamic University (IMSIU), Riyadh 13317, Saudi Arabia.

Email: emelsokkary@imamu.edu.sa

ORCID: https://orcid.org/0000-0003-3606-0112

Corresponding author: Emadeldin M. Elsokkary (emelsokkary@imamu.edu.sa)

Abstract

Rapid digital transformation in Saudi Arabia has intensified young adults' day-to-day reliance on smartphones, increasing the need for balanced, culturally appropriate measures of perceived digital well-being. This study aimed to psychometrically validate the Arabic version of the Perceived Digital Well-Being Scale (PDWS) among Saudi young adults aged (18–25). A cross-sectional instrument-validation study was conducted with (N=450) participants who were randomly split into two independent subsamples: Sample (1) for exploratory factor analysis (EFA) (n=225) and Sample (2) for confirmatory factor analysis (CFA) and convergent validity testing (n=225). EFA supported a clear three-factor structure (Emotional, Social, Cognitive). CFA confirmed a second-order model with excellent fit: $\chi^2(116)=133.275$, p=.130, $\chi^2/df=(1.149)$, CFI=(.986), TLI=(.983), RMSEA=(.026), and SRMR=(.055). Convergent validity was supported by positive associations with life satisfaction (SWLS) and negative associations with fear of missing out (FoMO), with the same pattern observed across both subsamples (Sample (2): SWLS r=(.416–.550), p<.01; FoMO r=(-.321–-.254), p<.01). Internal consistency was acceptable across subsamples (Cronbach's α=(.759–.886); McDonald's ω=(.761–.889)). Overall, the Arabic PDWS demonstrates robust structural validity, theoretically

consistent convergent validity, and adequate reliability, supporting its use as a multidimensional measure of perceived smartphone-related digital well-being among Saudi emerging adults.

Keywords: Perceived Digital Well-Being; Scale Validation; Saudi Young Adults; Fear of Missing Out; Life Satisfaction.

Introduction

Digital technology has become the default setting for how many young people learn, socialize, and manage daily life. In Saudi Arabia, this shift has unfolded alongside rapid national digital transformation and widespread adoption of smartphones and social platforms, making digitally mediated routines especially salient in the lives of young adults. Yet the psychological significance of this environment is still not fully captured by much of the existing literature, which has often been dominated by risk-focused outcomes such as problematic smartphone use and addiction-like patterns [1]. What remains comparatively underdeveloped—particularly in non-Western settings—is a balanced, person-centered understanding of digital well-being: how individuals themselves evaluate whether their digital engagement supports or undermines their functioning [2–5].

A major reason for this gap is conceptual and measurement inconsistency. "Digital well-being" has been used to refer to very different ideas—ranging from time-management and self-control to emotional strain, social connection, cognitive overload, and perceived life balance [2–5]. In practice, simply quantifying exposure (e.g., hours online) rarely tells us whether technology is experienced as helpful or harmful in daily life, and evidence suggests that links between digital engagement and well-being are often small on average and can vary across people, contexts, and developmental periods [6–9]. A more informative approach is to assess the perceived impact of digital engagement on key domains of functioning. This perspective aligns with recent measurement work that treats digital well-

being as a subjective appraisal of digital life across emotional, social, and cognitive spheres [10].

The Saudi context strengthens the case for such an approach. Social life is strongly relationship-oriented, and digital communication is woven into everyday obligations and expectations. In settings where relational closeness and responsiveness carry social meaning, technology can become a source of pressure rather than convenience: messages can imply an expectation of timely replies, group chats can create continuous social "presence," and delays in response may be interpreted as disengagement. These culturally shaped dynamics can turn everyday connectivity into a subtle burden—especially for young adults navigating social identity, peer evaluation, and boundary-setting. In other words, the same tools that facilitate closeness may also intensify social demand, thereby shaping how digital engagement is appraised in terms of well-being [2,3].

The present study focuses on Saudi young adults aged (18–25). This age range corresponds closely to what Arnett (2000) described as emerging adulthood, a developmental period characterized by identity exploration and intensive negotiation of social roles and relationships. The choice of (18–25) also addresses an important methodological and interpretive issue. National youth statistics often use broader categories (e.g., "youth" spanning mid-adolescence through the early thirties), which are valuable for population description but can combine markedly different developmental stages and life contexts. In contrast, concentrating on (18–25) provides a more developmentally coherent group in which digitally mediated life is especially central to peer connection, identity expression, and daily routines—while also reducing age-related heterogeneity that can obscure factor structure and validity patterns in psychometric testing [11].

Against this background, the Perceived Digital Well-Being Scale (PDWS) offers a focused solution to a persistent measurement problem: how to capture the lived balance of benefits and costs in digital life without presuming that technology is uniformly "good" or "bad." The PDWS is grounded in the idea that digital engagement can simultaneously support connection and productivity while also straining attention, emotions, or social comparison. It builds on earlier work that operationalized perceived digital well-being across emotional, social, and cognitive domains [10]. In a recent psychometric validation, Vera Cruz et al. (2025) examined the PDWS in young adult samples from the United States and the United Kingdom and reported evidence consistent with a stable multidimensional structure and theoretically meaningful correlates [12]. These findings are promising, but they do not resolve a central question for research in Arabic-speaking settings: will the same measurement structure and validity evidence hold in Saudi young adults, where cultural norms, communication practices, and digitally mediated social obligations may influence how items are understood and endorsed?

From a psychometric standpoint, scale use in a new linguistic and cultural context requires more than translation. The Standards for Educational and Psychological Testing emphasize that validity is a cumulative argument supported by multiple sources of evidence aligned with intended score interpretations [13]. Similarly, the International Test Commission's test adaptation guidelines stress careful translation procedures, documentation of decisions, and evaluation of measurement equivalence in the target population [14]. Modern measurement frameworks further highlight that structural validity, reliability, and hypothesis testing should be examined transparently and interpreted in relation to a construct's expected nomological network [15,16]. These principles are particularly relevant for perceived digital well-being, because culturally patterned

expectations about responsiveness, privacy, and social participation can shape both technology behavior and the perceived meaning of that behavior [2–4].

Consistent with these standards, the current study evaluates the PDWS factor structure in Saudi young adults by testing theoretically plausible competing models. At minimum, the PDWS can be conceptualized as correlated first-order domains (emotional, social, and cognitive), but it is also plausible that these domains reflect a broader higher-order perceived digital well-being factor. Because the PDWS is intended to capture an overarching appraisal of digital life across domains, examining both a correlated three-factor model and a second-order model provides a rigorous test of structural validity while remaining faithful to the measure's conceptual foundation [10,12].

To strengthen validity evidence beyond structure, the present study places PDWS scores within a focused nomological network using two convergent benchmarks: Fear of Missing Out (FoMO) and life satisfaction. Przybylski et al. (2013) defined FoMO as a persistent apprehension that others might be having rewarding experiences from which one is absent, coupled with a desire to stay continually connected [17]. Conceptually, FoMO can push digital engagement toward anxious monitoring, heightened social comparison, and difficulty disengaging—conditions that are likely to be experienced as draining rather than supportive. Evidence syntheses indicate that FoMO is reliably linked to more problematic patterns of social networking use, supporting its relevance as a convergent benchmark [18]. Recent Saudi evidence also supports the local relevance of FoMO and shows meaningful associations with technology-related outcomes [19]. On these grounds, higher FoMO is expected to relate to lower perceived digital well-being.

Life satisfaction provides a complementary benchmark. It reflects a global cognitive evaluation of one's quality of life [20], and it is widely used as a stable indicator of evaluative

well-being. In Saudi Arabia, the Arabic version of the Satisfaction with Life Scale has demonstrated strong psychometric support [21]. If perceived digital well-being captures a meaningful appraisal of how digital life supports everyday functioning, it should show a positive association with life satisfaction in theoretically coherent directions. Together, FoMO and life satisfaction enable a concise but informative assessment of convergent validity: perceived digital well-being should align negatively with FoMO and positively with life satisfaction.

In sum, the present study aimed to psychometrically validate the Arabic version of the PDWS in Saudi young adults aged (18–25) by examining its factor structure and testing its associations with FoMO and life satisfaction. Establishing a robust Arabic PDWS would provide researchers with a culturally appropriate tool for studying digital well-being in Saudi Arabia and would support more cumulative, measurement-grounded research on how digital life is experienced and evaluated in this context.

Methods

Study Design and Setting

The present study employed a cross-sectional instrument-validation design to examine the psychometric properties of the Arabic version of the Perceived Digital Well-Being Scale (PDWS) in Saudi young adults aged (18–25). Data were collected via an anonymous online survey administered in Riyadh, Saudi Arabia, between (March 2025) and (May 2025). Participants completed the PDWS along with two convergent validity measures: Fear of Missing Out (FoMO) and life satisfaction.

Participants

A total of (N=450) Saudi young adults aged (18–25) participated in the study. Participants were randomly split into two equal subsamples: Sample 1 (EFA subsample; n=225) and Sample 2 (CFA subsample; n=225). Exploratory factor analysis (EFA) was conducted in Sample 1, confirmatory factor analysis (CFA) was conducted in Sample 2, and convergent validity correlations were examined in both subsamples.

In Sample 1 (EFA subsample), age ranged from (18) to (25) years (M=22.27, SD=1.87), with (110) females (48.9%) and (115) males (51.1%). Most participants were single (n=161, 71.6%), followed by married (n=57, 25.3%) and divorced/widowed (n=7, 3.1%). Regarding education, (152) participants reported a bachelor's degree (67.6%), (40) high school or less (17.8%), and (33) postgraduate education (14.7%).

In Sample 2 (CFA subsample), age ranged from (18) to (25) years (M=21.99, SD=1.92), with (114) females (50.7%) and (111) males (49.3%). Most participants were single (n=157, 69.8%), followed by married (n=58, 25.8%) and divorced/widowed (n=10, 4.4%). Education levels included bachelor's degree (n=148, 65.8%), high school or less (n=39, 17.3%), and postgraduate education (n=38, 16.9%).

Across the full sample, the mean age was (M=22.13, SD=1.90), with (224) females (49.8%) and (226) males (50.2%). Overall marital status was (318) single (70.7%), (115) married (25.6%), and (17) divorced/widowed (3.8%). Education was distributed as (300) bachelor's degree (66.7%), (79) high school or less (17.6%), and (71) postgraduate education (15.8%). No missing data were observed for the reported sociodemographic variables in either subsample.

Ethical Approval and Consent

The study protocol was reviewed and approved by the Institutional Review Board (IRB), Imam Mohammad Ibn Saud Islamic University, Riyadh, Saudi Arabia (IRB registration: HAPO-01-R-061; Project No. 244/2025; approval date: 10 March 2025; full review). All participants provided electronic informed consent prior to participation. Participation was voluntary and responses were collected anonymously.

Procedure

Participants were recruited through social media platforms in Saudi Arabia between 15 March 2025 and 30 May 2025. Data were collected using a self-administered online questionnaire created and distributed via Google Forms. Participation was fully anonymous; no identifying information (e.g., names, phone numbers, email addresses, or student IDs) was collected. No incentives or compensation were provided. The survey required approximately (10–15) minutes to complete.

Translation and Cultural Adaptation of the PDWS

The PDWS was translated and culturally adapted into Arabic using established cross-cultural test adaptation recommendations, with an emphasis on conceptual and semantic equivalence rather than literal translation [13,14]. First, two independent forward translations were produced by bilingual (Arabic–English) translators familiar with psychological and psychometric terminology. Second, a reconciliation meeting was conducted by the research team to compare the two Arabic versions item-by-item, resolve discrepancies, and agree on a single unified Arabic wording for each item, guided by clarity, accuracy of meaning, and suitability for Saudi young adults. Third, the unified Arabic version was reviewed by an expert panel (n=10) specializing in psychology and psychometrics to evaluate linguistic clarity, cultural appropriateness, and alignment with the

intended construct, resulting in minor wording refinements without changing the underlying meaning. Fourth, a blind back-translation into English was completed by an independent translator who had not accessed the original PDWS. The back-translated and original versions were compared to identify potential semantic deviations, and iterative adjustments were made to the Arabic items until satisfactory equivalence was achieved [22,23].

Measures

Perceived Digital Well-Being Scale (PDWS)

Perceived digital well-being was assessed using the Perceived Digital Well-Being Scale (PDWS), a (17)-item self-report measure comprising three domains: emotional digital well-being ((7) items), social digital well-being ((6) items), and cognitive digital well-being ((4) items) [12]. Each item is presented in a bipolar format reflecting negative versus positive perceived outcomes of smartphone use, with responses recorded on a (5)-point scale ranging from (1) at the left pole to (5) at the right pole; higher scores indicate higher perceived digital well-being. Domain scores were computed by averaging the corresponding items within each domain, and a total PDWS score was computed by averaging all (17) items. In the current study, based on the (EFA/CFA) results, item (S11) was included in the Emotional domain when computing domain scores (Emotional=8; Social=5; Cognitive=4).

Convergent Validity Measures

Satisfaction with Life Scale (SWLS)

To assess participants' global cognitive evaluation of life satisfaction, the Satisfaction with Life Scale (SWLS), originally developed by Diener et al. (1985), was used [20]. The present study employed the Arabic version validated for the Saudi Arabian context by Al-Dossary (2022) [21]. The SWLS is a (5)-item instrument rated on a (7)-point Likert-type scale ranging from (1=Strongly Disagree) to (7=Strongly Agree). Total scores range from

(5–35), with higher scores indicating greater life satisfaction. In the Saudi context, Al-Dossary (2022) reported high internal consistency reliability (Cronbach's alpha = .88 for students and .84 for employees, and McDonald's omega values were .88 and .84, respectively). Evidence of validity was supported by a robust one-factor CFA solution with good fit, expected associations with well-being and happiness, negative correlations with depression indices, and established measurement invariance across gender, marital status, and role (students vs. employees) [21].

Fear of Missing Out Scale (FoMO)

Fear of missing out was assessed using the FoMO Scale originally developed by Przybylski et al. (2013). This study employed an Arabic version supported by recent evidence from the Saudi context as reported by Al-Abyadh (2025) [19]. The FoMO Scale is a (10)-item self-report measure rated on a (5)-point Likert scale ranging from (1=Not at all true of me) to (5=Extremely true of me), with higher scores indicating higher levels of FoMO. Al-Abyadh (2025) reported satisfactory internal consistency in a Saudi sample and supported construct validity through theoretically consistent associations with related constructs, particularly social media addiction and psychological distress [19].

Results

Exploratory Factor Analysis (EFA) Results

Exploratory factor analysis was conducted on the EFA subsample (n=225) to examine the underlying factor structure of the Arabic PDWS. The data were suitable for factor analysis, as indicated by a high Kaiser–Meyer–Olkin (KMO) measure of sampling adequacy (.893) and a significant Bartlett's test of sphericity ($\chi^2(136)=1383.982$, $p<.001$). The EFA

was performed using maximum likelihood extraction with an oblique rotation (Direct Oblimin with Kaiser normalization).

Both the Kaiser criterion (eigenvalues>1) and inspection of the scree plot supported a three-factor solution. The first three eigenvalues were (5.641), (2.440), and (1.175), respectively. Based on extraction sums of squared loadings, the three retained factors accounted for a cumulative (45.614%) of the extracted variance. The maximum likelihood goodness-of-fit test further supported the adequacy of the three-factor solution ($\chi^2(88)=82.597$, $p=.643$). Rotation converged in (6) iterations.

The rotated pattern matrix yielded a clear and interpretable structure with no salient cross-loadings, using a loading cutoff of (.30). Factor (1) consisted primarily of the emotional domain items (E1–E7), with loadings ranging from (.471–.689). Factor (2) consisted of the cognitive domain items (C14–C17), with strong loadings ranging from (.765–.838). Factor (3) consisted of the social domain items (S8–S10, S12–S13), with loadings ranging from (.320–.710). Item (S11) loaded on Factor (1) (loading=.715).

Table 1. *KMO and Bartlett's Test of Sphericity (EFA Subsample, n=225)*

**Test/Index**	**Value**
Kaiser–Meyer–Olkin (KMO)	.893
Bartlett's test of sphericity: χ^2	1383.982
df	136
p	<.001

Table 2. *Rotated Pattern Matrix (Direct Oblimin) for the Arabic PDWS With Initial Eigenvalues and Extracted Variance (EFA Subsample, n=225)*

**Item**	**Factor (1)**	**Factor (2)**	**Factor (3)**
E1	.537	—	—
E2	.689	—	—
E3	.597	—	—
E4	.490	—	—

Item	Factor (1)	Factor (2)	Factor (3)
E5	.501	—	—
E6	.616	—	—
E7	.471	—	—
S8	—	—	.694
S9	—	—	.320
S10	—	—	.673
S11	.715	—	—
S12	—	—	.710
S13	—	—	.433
C14	—	.822	—
C15	—	.765	—
C16	—	.806	—
C17	—	.838	—
**Initial eigenvalue**	5.641	2.440	1.175
**% Variance extraction**	28.088	13.927	3.599

Note. *Extraction method was maximum likelihood. Rotation method was Direct Oblimin with Kaiser normalization. Rotation converged in (6) iterations. "—" indicates coefficients below the reporting cutoff (.30). No salient cross-loadings were observed. The cumulative extracted variance explained by the three-factor solution was (45.614%).*

Confirmatory Factor Analysis (CFA) Results

Confirmatory factor analysis (CFA) was conducted on the CFA subsample (n=225) using (AMOS 26) to verify the factor structure of the Arabic PDWS. The model was estimated using maximum likelihood (ML). A second-order model was specified in which Perceived Digital Well-Being was modeled as a higher-order construct reflected by three first-order dimensions (Emotional, Social, and Cognitive). The model demonstrated good fit to the data: $\chi^2(116)=133.275$, $p=.130$, with $\chi^2/df=(1.149)$. Incremental fit indices indicated excellent fit (CFI=.986, TLI=.983, IFI=.986, NFI=.901), and residual-based fit was acceptable (SRMR=.055). The RMSEA also indicated excellent fit (RMSEA=.026, (90%) CI [.000, .044], PCLOSE=.990).

All standardized factor loadings were statistically significant ($p<.001$). Standardized loadings ranged from (.422–.683) for the Emotional dimension, (.574–.731) for the Social dimension, and (.738–.791) for the Cognitive dimension. Consistent with the EFA results, item (S11) loaded on the Emotional factor (standardized loading=.603, $p<.001$). Squared multiple correlations (R^2) indicated that the indicators explained meaningful proportions of variance, with R^2 values ranging from (.178–.467) for Emotional items, (.330–.534) for Social items, and (.545–.626) for Cognitive items.

At the second-order level, the higher-order PDWS factor showed strong standardized loadings on the three first-order dimensions (Emotional=.894, Social=.749, Cognitive=.759), all statistically significant ($p<.001$), supporting the interpretation of PDWS as a higher-order construct reflected by the three domains.

Table 3. *Confirmatory Factor Analysis Model Fit Indices for the Second-Order PDWS Model (CFA Subsample, n=225)*

**Fit index**	**Value**
χ^2 (df)	133.275 (116)
p	.130
χ^2/df	1.149
CFI	.986
TLI	.983
IFI	.986
NFI	.901
SRMR	.055
RMSEA (90% CI)	.026 [.000, .044]
PCLOSE	.990

Note*. The model was estimated using maximum likelihood (ML) in (AMOS 26). RMSEA confidence interval represents the (90%) CI reported by AMOS.*

Table 4. *Standardized Factor Loadings and Squared Multiple Correlations (R^2) for PDWS Items (Second-Order CFA; CFA Subsample, n=225)*

First-order factor	Item	Standardized loading	R^2 (SMC)
Emotional	E1	.498	.248
	E2	.683	.467
	E3	.620	.384
	E4	.466	.217
	E5	.652	.426
	E6	.567	.321
	E7	.422	.178
	S11	.603	.364
Social	S8	.724	.525
	S9	.574	.330
	S10	.671	.450
	S12	.621	.386
	S13	.731	.534
Cognitive	C14	.738	.545
	C15	.783	.613
	C16	.791	.626
	C17	.766	.586

Note*. All standardized loadings were statistically significant ($p<.001$). R^2 values are squared multiple correlations (SMC) reported by AMOS.*

Table 5*. Standardized Second-Order Loadings of PDWS on the First-Order Dimensions (CFA Subsample, n=225)*

Higher-order factor	First-order dimension	Standardized loading	R^2 (SMC)
Perceived Digital Well-Being	Emotional	.894	.799
	Social	.749	.561
	Cognitive	.759	.576

Note*. Second-order loadings are standardized estimates from the second-order CFA model. All second-order loadings were statistically significant ($p<.001$). R^2 values are squared multiple correlations reported by AMOS.*

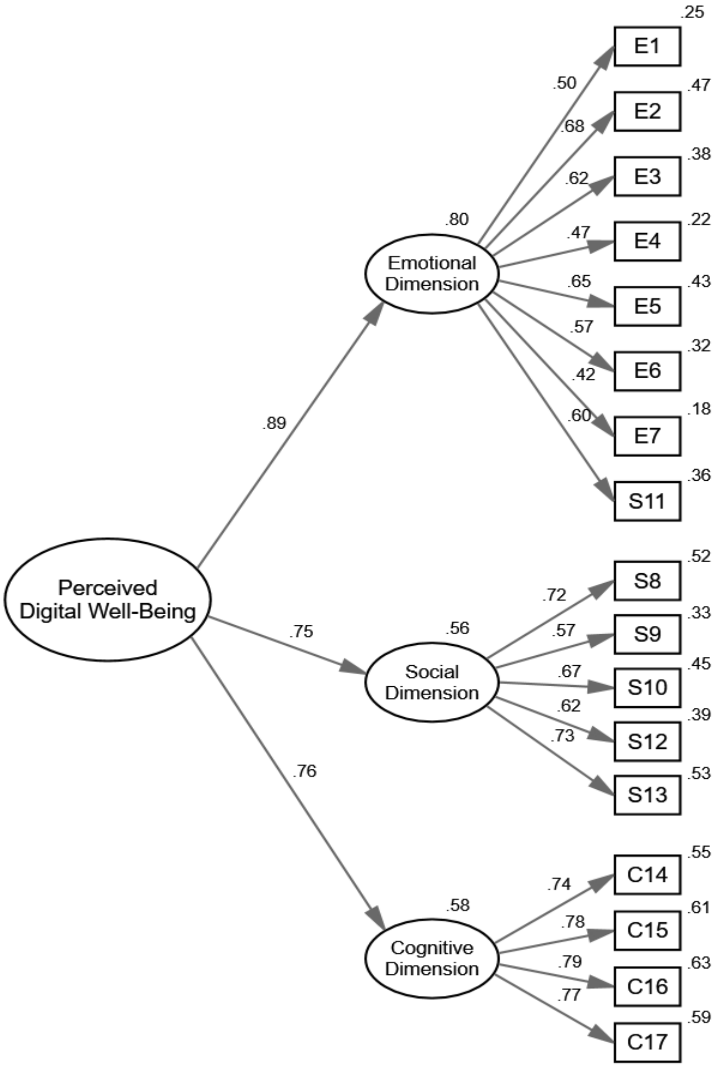

Figure 1. *Standardized second-order CFA model of the Arabic PDWS (n=225). Standardized loadings are shown on paths; values above items are* R^2.

Convergent Validity (SWLS and FoMO)

Convergent validity of the Arabic PDWS was examined by correlating PDWS scores with (SWLS) and (FoMO) separately in Sample 1 (EFA subsample; n=225) and Sample 2 (CFA subsample; n=225). In Sample 1, PDWS domain scores and the total PDWS score were positively associated with life satisfaction (SWLS) (r range = .296–.432, $p \leq .01$) and negatively associated with fear of missing out (FoMO) (r range = -.141 to -.208, $p \leq .05$). In Sample 2, the same pattern was replicated with larger magnitudes: PDWS scores correlated

positively with SWLS (r range = .416–.550, p<.01) and negatively with FoMO (r range = -.254 to -.321, p<.01). Overall, these findings support convergent validity by demonstrating theoretically consistent associations across two independent subsamples.

Table 6. *Convergent Validity Correlations Between PDWS Scores, SWLS, and FoMO Across Two Independent Subsamples*

**PDWS**	**Sample 1 (EFA; n=225)**		**Sample 2 (CFA; n=225)**	
	**SWLS**	**FoMO**	**SWLS**	**FoMO**
Emotional	.353**	-.141*	.453**	-.254**
Social	.361**	-.189**	.416**	-.269**
Cognitive	.296**	-.162*	.495**	-.271**
PDWS	.432**	-.208**	.550**	-.321**

Note. *Pearson correlations are reported. * p<.05. ** p<.01 (two-tailed).*

Reliability

Internal consistency reliability of the Arabic PDWS was evaluated in both subsamples using Cronbach's alpha (α) and McDonald's omega (ω). In Sample 1 (EFA subsample; n=225), reliability estimates were acceptable to excellent across the three dimensions (α range = .759–.885; ω range = .761–.889), and for the total PDWS score (α=.872; ω=.861). In Sample 2 (CFA subsample; n=225), internal consistency was similarly acceptable across dimensions (α range = .788–.850; ω range = .787–.851) and was strong for the total score (α=.886; ω=.883). Overall, these results indicate that the Arabic PDWS yields reliable scores at both the domain and total-score levels across two independent subsamples.

Table 7. *Internal Consistency Reliability (Cronbach's α and McDonald's ω) for the PDWS Across Two Independent Subsamples*

PDWS	Items (k)	Sample 1 (EFA; n=225) Cronbach's α	McDonald's ω	Sample 2 (CFA; n=225) Cronbach's α	McDonald's ω
Emotional	(8)	.822	.822	.788	.787
Social	(5)	.759	.761	.797	.799
Cognitive	(4)	.885	.889	.850	.851
PDWS	(17)	.872	.861	.886	.883

Note. *Cronbach's α and McDonald's ω are reported for each dimension and for the total PDWS score.*

Construct Reliability and Validity

Construct-level reliability and validity of the Arabic PDWS were evaluated in the CFA subsample (Sample 2; n=225) using composite reliability (CR), average variance extracted (AVE), and the Fornell–Larcker criterion. As shown in Table (8), CR values indicated good construct reliability across the three dimensions (CR range = .790–.853). Discriminant validity was partially supported according to the Fornell–Larcker criterion (Fornell & Larcker, 1981).

Table 8. *Construct Reliability and Validity (Sample 2; n=225)*

Construct	CR	AVE	(1) Emotional	(2) Social	(3) Cognitive
**(1) Emotional**	.790	.326	**.571**		
**(2) Social**	.799	.445	.669	**.667**	
**(3) Cognitive**	.853	.593	.678	.568	**.770**

Note. *CR = composite reliability; AVE = average variance extracted; Diagonal values are √AVE; off-diagonal values are standardized latent factor correlations from AMOS.*

Discussion

The present study provides initial evidence that the Arabic version of the Perceived Digital Well-Being Scale (PDWS) is a psychometrically sound instrument for assessing Saudi young adults' perceived smartphone-related well-being. Using a split-sample design,

the exploratory results supported a clear three-factor structure corresponding to the Emotional, Social, and Cognitive dimensions, and the confirmatory results further supported a hierarchical conceptualization in which these three domains are explained by an overarching perceived digital well-being factor. This pattern is consistent with the scale's theoretical premise that individuals form domain-specific appraisals of smartphone use (e.g., emotions, relationships, and day-to-day functioning) that cohere into a broader subjective evaluation of digital well-being [10,12].

From a structural standpoint, sampling adequacy and factorability indicators were strong, supporting the suitability of the item set for latent structure examination. The EFA results aligned with the intended three-domain solution, and item loadings exceeded the adopted cutoff, with no evidence of problematic cross-loadings. The CFA results provided further support for the measurement model, with fit indices indicating excellent global fit (e.g., CFI=.986, RMSEA=.026, SRMR=.055, and CMIN/DF=1.149), consistent with commonly cited criteria for well-fitting CFA models [24]. Importantly, the second-order specification was empirically justified by the strong loadings of the three first-order factors on the general PDWS factor, supporting the use of both domain scores and a total PDWS score when the research aim concerns overall perceived digital well-being.

A notable finding was that the item reflecting the perceived impact of smartphone use on "true friendships" (S11) aligned more strongly with the Emotional dimension in the Saudi sample, rather than the Social dimension as originally proposed. Conceptually, this pattern is defensible because appraisals about the authenticity of friendships may function primarily as affect-laden evaluations (e.g., feelings of reassurance, insecurity, comfort, or emotional safety) rather than as a purely interactional indicator of social contact frequency or connection. In relationally oriented settings, where the quality of social bonds is central to emotional stability and day-to-day affect, reflections on "true friendships" may be

experienced as an emotional consequence of smartphone use (e.g., worry, calmness, satisfaction, or distress), thereby clustering with other affective outcomes. Consistent with this item-level behavior, domain scoring and reliability estimation in the present study reflected the empirically supported allocation of S11 to the Emotional domain. This culturally plausible interpretation does not undermine the model; instead, it illustrates the value of cross-cultural validation in testing whether item–factor alignment remains stable across contexts, as recommended in test adaptation guidance [14].

Convergent validity evidence was also consistent with theory. PDWS scores—both at the domain and total levels—showed positive associations with life satisfaction as measured by the SWLS, indicating that more favorable perceived smartphone-related well-being is linked to higher global cognitive evaluations of one's life [20,21]. At the same time, PDWS scores were negatively associated with FoMO, suggesting that individuals who experience greater fear of missing out tend to report poorer perceived digital well-being, in line with FoMO models emphasizing persistent social-monitoring concerns and their psychological costs [17,19]. The replication of these associations across both subsamples strengthens confidence that the nomological network of the Arabic PDWS aligns with theoretically relevant constructs.

Regarding reliability, internal consistency estimates were acceptable to strong for the PDWS domains and the total score across both subsamples, with Cronbach's alpha and McDonald's omega largely converging—an encouraging pattern given that omega is often preferred when congeneric measurement is expected. In addition, construct reliability indicators derived from the CFA model (CR) supported adequate construct-level reliability. AVE values were mixed across domains, and discriminant validity was only partially supported under the Fornell–Larcker criterion, suggesting that some overlap among domains may reflect shared variance attributable to the broader higher-order construct [25,26]. In this

regard, presenting both internal consistency (alpha/omega) and model-based reliability/validity indices (CR/AVE) provides a transparent evaluation of measurement quality, consistent with contemporary psychometric reporting expectations in scale validation work [15].

Overall, the findings support the Arabic PDWS as a practical tool for assessing perceived smartphone-related well-being among Saudi young adults, offering researchers and practitioners a balanced measurement approach that moves beyond a sole focus on problematic use and toward understanding how individuals perceive the benefits and costs of smartphone engagement across emotional, social, and cognitive life domains [10,12,27].

Limitations and Future Directions

Several limitations should be acknowledged. First, participants were recruited online through social media and completed the survey anonymously via Google Forms. Although this approach enabled efficient access to the target age group, it may have introduced self-selection bias and limits the representativeness of the sample. Second, the study was conducted in Riyadh and focused on Saudi young adults aged (18–25); therefore, generalization to other regions, age groups, or Arab populations should be made cautiously. Third, the cross-sectional design precludes causal conclusions regarding the observed associations between perceived digital well-being, FoMO, and life satisfaction. Fourth, reliability evidence in the present study was limited to internal consistency; future work should examine temporal stability using test–retest designs. Finally, additional validity evidence is warranted, including measurement invariance across key groups (e.g., gender, education level) and predictive validity in relation to theoretically relevant outcomes that capture both adaptive and strain-related aspects of digital experience (e.g., sleep quality, academic/work performance, psychological distress, and digital stress; [28]). The placement

of item (S11) on the Emotional domain also highlights the importance of continued evaluation of item functioning across contexts, including replication in broader Saudi samples and across other Arabic-speaking populations.

Conclusion

This study provides initial psychometric support for the Arabic version of the Perceived Digital Well-Being Scale (PDWS) among Saudi young adults aged (18–25). Using a split-sample approach, the findings supported a clear three-domain structure and a well-fitting second-order model, indicating that emotional, social, and cognitive appraisals of smartphone use coherently reflect an overarching perceived digital well-being construct. The Arabic PDWS demonstrated theoretically consistent convergent validity through positive associations with life satisfaction (SWLS) and negative associations with Fear of Missing Out (FoMO), and it showed acceptable internal consistency across domains and the total score in two independent subsamples. Future research should extend this evidence by examining temporal stability, measurement invariance across key groups, and predictive validity for relevant outcomes in Saudi and other Arabic-speaking contexts.

Data Availability Statement

All data underlying the findings described in this manuscript are publicly available in Zenodo (doi: 10.5281/zenodo.18882971).

Conflicts of Interest Statement

The author declares no conflict of interest.

References

1. Al-Mohaimeed A, Alharbi M, Mahmud I. Prevalence and Associated Factors of Problematic Use of Smartphones Among Adults in Qassim, Saudi Arabia: Cross-sectional Survey. JMIR Public Health Surveill. 2022;8:e37451. https://doi.org/10.2196/37451

2. Vanden Abeele MMP. Digital Wellbeing as a Dynamic Construct. Commun Theory. 2021;31:932–55. https://doi.org/10.1093/ct/qtaa024

3. Vanden Abeele MMP, Nguyen MH. Digital well-being in an age of mobile connectivity: An introduction to the Special Issue. Mob Media Commun. 2022;10:174–89. https://doi.org/10.1177/20501579221080899

4. OECD. How's Life for Children in the Digital Age? [Internet]. OECD Publishing; 2025 [cited 2026 Mar 5]. https://doi.org/10.1787/0854b900-en

5. Büchi M. Digital well-being theory and research. New Media Soc. 2024;26:172–89. https://doi.org/10.1177/14614448211056851

6. Orben A, Przybylski AK. The association between adolescent well-being and digital technology use. Nat Hum Behav. 2019;3:173–82. https://doi.org/10.1038/s41562-018-0506-1

7. Valkenburg PM, Meier A, Beyens I. Social media use and its impact on adolescent mental health: An umbrella review of the evidence. Curr Opin Psychol. 2022;44:58–68. https://doi.org/10.1016/j.copsyc.2021.08.017

8. Vuorre M, Orben A, Przybylski AK. There Is No Evidence That Associations Between Adolescents' Digital Technology Engagement and Mental Health Problems Have Increased. Clin Psychol Sci. 2021;9:823–35. https://doi.org/10.1177/2167702621994549

9. Vuorre M, Przybylski AK. Global Well-Being and Mental Health in the Internet Age. Clin Psychol Sci. 2024;12:917–35. https://doi.org/10.1177/21677026231207791

10. Rosič J, Carbone L, Vanden Abeele MMP, Lobe B, Vandenbosch L. Measuring digital well-being in everyday life among Slovenian adolescents: The Perceived Digital Well-Being in Adolescence Scale. J Child Media. 2024;18:99–119. https://doi.org/10.1080/17482798.2023.2272651

11. Arnett JJ. Emerging adulthood: A theory of development from the late teens through the twenties. Am Psychol. 2000;55:469–80. https://doi.org/10.1037/0003-066X.55.5.469

12. Vera Cruz G, Liberacka-Dwojak M, Wiłkość-Dębczyńska M, Aktaş Terzioğlu M, Farchione T, Lecomte T, et al. Perceived Digital Well-Being Scale in the United States and United Kingdom: Psychometric Validation Study. JMIR Ment Health. 2025;12:e78334–e78334. https://doi.org/10.2196/78334

13. American Educational Research Association, American Psychological Association, National Council on Measurement in Education. Standards for educational and psychological testing [Internet]. American Educational Research Association; 2014. https://www.testingstandards.net/uploads/7/6/6/4/76643089/standards_2014edition.pdf

14. International Test Commission. The ITC guidelines for translating and adapting tests [Internet]. 2nd ed. 2017. https://www.intestcom.org/files/guideline_test_adaptation_2ed.pdf

15. Mokkink LB, Terwee CB, Patrick DL, Alonso J, Stratford PW, Knol DL, et al. The COSMIN checklist for assessing the methodological quality of studies on measurement properties of health status measurement instruments: an international Delphi study. Qual Life Res. 2010;19:539–49. https://doi.org/10.1007/s11136-010-9606-8

16. Prinsen CAC, Mokkink LB, Bouter LM, Alonso J, Patrick DL, De Vet HCW, et al. COSMIN guideline for systematic reviews of patient-reported outcome measures. Qual Life Res. 2018;27:1147–57. https://doi.org/10.1007/s11136-018-1798-3

17. Przybylski AK, Murayama K, DeHaan CR, Gladwell V. Motivational, emotional, and behavioral correlates of fear of missing out. Comput Hum Behav. 2013;29:1841–8. https://doi.org/10.1016/j.chb.2013.02.014

18. Fioravanti G, Casale S, Benucci SB, Prostamo A, Falone A, Ricca V, et al. Fear of missing out and social networking sites use and abuse: A meta-analysis. Comput Hum Behav. 2021;122:106839. https://doi.org/10.1016/j.chb.2021.106839

19. Al-Abyadh MHA. The fear of missing out and social media addiction: A cross-sectional and quasi-experimental approach. Heliyon. 2025;11:e41958. https://doi.org/10.1016/j.heliyon.2025.e41958

20. Diener E, Emmons RA, Larsen RJ, Griffin S. The Satisfaction With Life Scale. J Pers Assess. 1985;49:71–5. https://doi.org/10.1207/s15327752jpa4901_13

21. Al-Dossary, S. Satisfaction with life scale in Saudi Arabia: Psychometric properties, measurement invariance, and latent means differences across sex, marital status, and between employees and students. Saudi Arabia: Saudi Journal of Psychological Sciences; 2022;29–41.

22. Beaton DE, Bombardier C, Guillemin F, Ferraz MB. Guidelines for the Process of Cross-Cultural Adaptation of Self-Report Measures: Spine. 2000;25:3186–91. https://doi.org/10.1097/00007632-200012150-00014

23. Sousa VD, Rojjanasrirat W. Translation, adaptation and validation of instruments or scales for use in cross-cultural health care research: a clear and user-friendly guideline. J Eval Clin Pract. 2011;17:268–74. https://doi.org/10.1111/j.1365-2753.2010.01434.x

24. Hu L, Bentler PM. Cutoff criteria for fit indexes in covariance structure analysis: Conventional criteria versus new alternatives. Struct Equ Model Multidiscip J. 1999;6:1–55. https://doi.org/10.1080/10705519909540118

25. Fornell C, Larcker DF. Evaluating Structural Equation Models with Unobservable Variables and Measurement Error. J Mark Res. 1981;18:39–50. https://doi.org/10.1177/002224378101800104

26. Raykov T. Estimation of Composite Reliability for Congeneric Measures. Appl Psychol Meas. 1997;21:173–84. https://doi.org/10.1177/01466216970212006

27. Chen S, Ebrahimi OV, Cheng C. New Perspective on Digital Well-Being by Distinguishing Digital Competency From Dependency: Network Approach. J Med Internet Res. 2025;27:e70483. https://doi.org/10.2196/70483

28. Funke C, Rothert-Schnell C, Walsh G, Mangiò F, Pedeliento G, Takahashi I. The digital stress scale: cross-cultural application, validation, and development of a short scale. Rev Manag Sci [Internet]. 2025 [cited 2026 Mar 5]; https://doi.org/10.1007/s11846-025-00906-w

Supporting Information

S1 Appendix. Arabic Perceived Digital Well-Being Scale (PDWS) (CC BY 4.0).

S2 Appendix. PDWS English version (CC BY 4.0).

Figure

Click here to access/download;Figure;Figure 1.jpeg

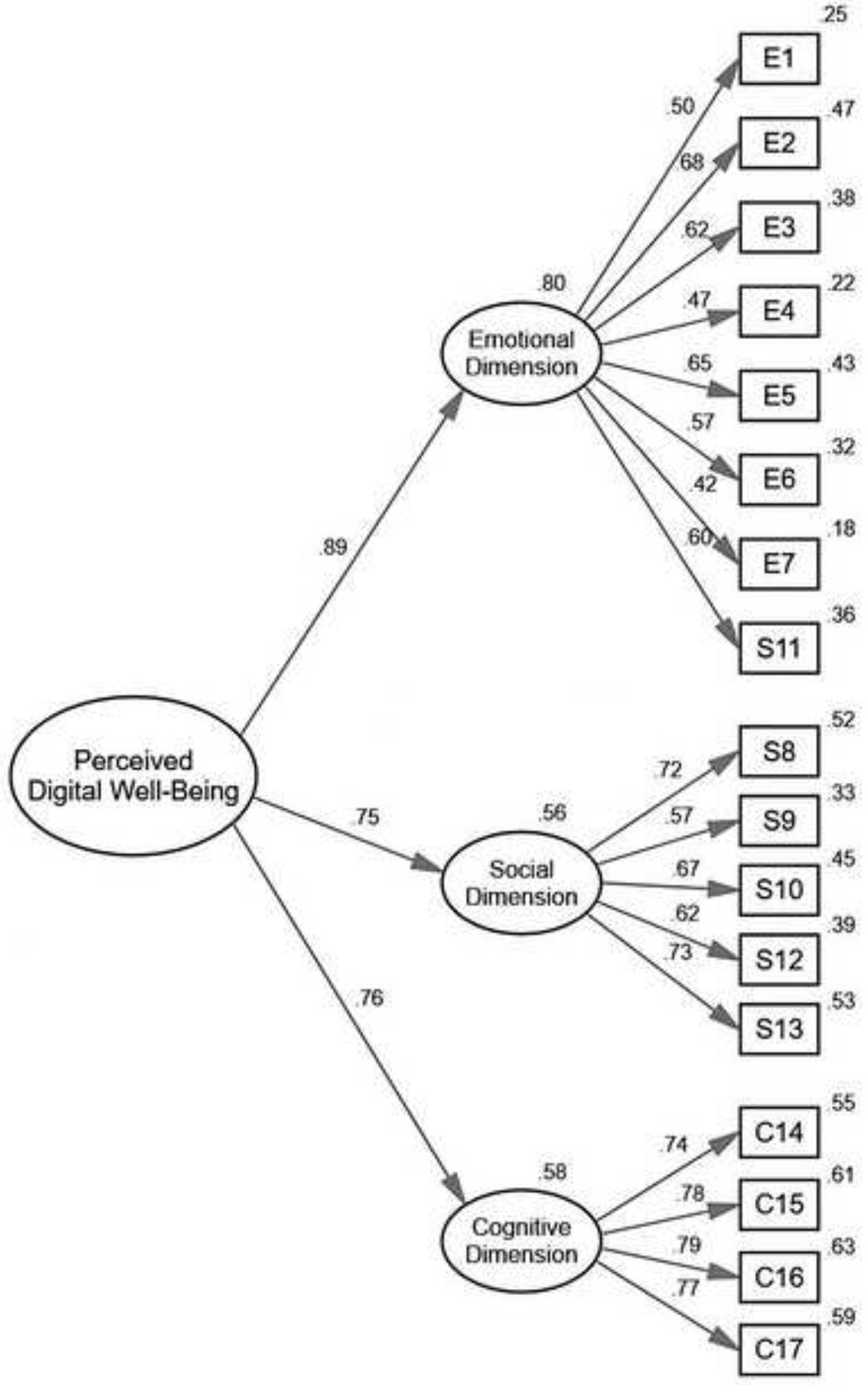

Click here to access/download
Supporting Information
S1_Appendix_Arabic_PDWS.pdf

Click here to access/download
Supporting Information
S2 Appendix. PDWS English version.pdf
