# Supplementary material for: Psychometric properties of the Arabic Perceived Digital Well-Being Scale (PDWS) in Saudi young adults
Source: PLoS One. 2026 Jul 30;21(7):e0354474. doi: 10.1371/journal.pone.0354474 (PMC13422880; doi:10.1371/journal.pone.0354474)
Supplement: Attachment — Submitted filename: Response_to_Reviewers_PONE-D-26-11267.docx [file pone.0354474.s004.docx]

**Response to Reviewers**

PLOS ONE – Manuscript PONE-D-26-11267

*Psychometric Properties of the Arabic Perceived Digital Well-Being Scale (PDWS) in Saudi Young Adults*

**Journal Requirements**

**JR1. PLOS ONE Style Requirements**

*Please ensure that your manuscript meets PLOS ONE's style requirements, including those for file naming.*

**Response:**

The revised manuscript has been checked against PLOS ONE style requirements, including file naming, line numbering, figure handling, supporting information files, and final formatting. The manuscript has been prepared with continuous line numbering, double-spaced text, sentence-case headings, in-text citation to Fig 1, and separate upload of the figure file as Fig1.tif.

**JR2. Role of Funders**

*Please state what role the funders took in the study. If the funders had no role, please state: "The funders had no role in study design, data collection and analysis, decision to publish, or preparation of the manuscript". If this statement is not correct you must amend it as needed .*

*Please include this amended Role of Funder statement in your cover letter; we will change the online submission form on your behalf*

**Response:**

The Role of Funders statement has been added as requested. The funding statement will also be included in the cover letter and updated in the submission system as follows: "This work was supported and funded by the Deanship of Scientific Research at Imam Mohammad Ibn Saud Islamic University (IMSIU) (grant number IMSIU-DDRSP2601). The funders had no role in study design, data collection and analysis, decision to publish, or preparation of the manuscript."

**JR3. Data Availability Statement**

*3. Please note that your Data Availability Statement is currently missing the repository name and/or the DOI/accession number of each dataset OR a direct link to access each database. If your manuscript is accepted for publication, you will be asked to provide these details on a very short timeline. We therefore suggest that you provide this information now, though we will not hold up the peer review process if you are unable.*

**Response:**

I updated the Data Availability Statement to explicitly include the repository name and DOI. All data underlying the findings described in this manuscript are publicly available in Zenodo (doi: 10.5281/zenodo.18882971).

**JR4. In-Text Reference to Fig 1**

*Please ensure that you refer to Fig 1 in your text as, if accepted, production will need this reference to link the reader to the figure.*

**Response:**

I added an explicit in-text reference to Fig 1 in the CFA Results section. The manuscript now states: "The standardized path diagram for the second-order CFA model is shown in Fig 1." This ensures that the figure is clearly linked within the manuscript text.

**JR5. Figures as Separate TIFF/EPS Files**

*Please remove your figures from within your manuscript file, leaving only the individual TIFF/EPS image files, uploaded separately. These will be automatically included in the reviewers’ PDF.*

**Response:**

Fig 1 has been prepared as a separate high-resolution TIFF file and will be uploaded separately as Fig1.tif in accordance with the journal’s requirements. The figure image has been removed from the manuscript body, leaving the in-text citation, insertion marker, and figure caption in the manuscript.

**JR6. In-Text References to Tables 1–7**

*We note you have included a table to which you do not refer in the text of your manuscript. Please ensure that you refer to Table 1-7 in your text; if accepted, production will need this reference to link the reader to the Table.*

**Response:**

I reviewed the revised manuscript and updated the in-text table references to ensure that each table is explicitly cited before or at the point where it is introduced. Because a new sociodemographic table was added in response to Reviewer 1, Comment 2.5, the revised manuscript now includes Tables 1–11. I added or revised the immediate in-text references to Tables 2–8, standardized the reference to Table 9, and verified that Table 1 and Tables 10–11 were already explicitly cited in the appropriate sections. All table numbering and in-text references have now been checked for consistency throughout the manuscript.

**Reviewer 1 Comments**

**Comment 1.1 – Origin and Prior Evidence of the PDWS**

*Explicitly state the origin of the PDWS instrument and its previous evidence, including content validity, internal and external structure, and adaptations in other contexts, among others.*

**Response:**

Thank you for this helpful comment. I revised the Introduction to state more explicitly the origin of the PDWS and the prior evidence supporting the instrument. Specifically, I clarified that the PDWS originates from the Perceived Digital Well-Being in Adolescence Scale (PDWBA), developed by Rosič et al. to assess adolescents’ subjective perceptions of whether the benefits of smartphone use outweigh its drawbacks across emotional, social, and cognitive domains. I also added that the original instrument included 17 bipolar items and was developed through a scale-development process involving a multidisciplinary team, providing content-related support for the item pool and initial evidence for the internal three-domain structure. In addition, I clarified that Vera Cruz et al. later examined an adapted young-adult version of the instrument, renamed the PDWS, in samples from the United States and the United Kingdom, with evidence supporting its three-factor structure, internal consistency, convergent validity, and measurement invariance across national samples. This revision better situates the present Arabic validation study within the existing psychometric literature and clarifies the need for validation in Arabic-speaking Saudi young adults.

**Comment 1.2 – National Population Census Statistics**

*Include demographic statistics (i.e., National Population Census) regarding the age range of the study’s target population in this country.*

**Response:**

Thank you for this suggestion. I revised the Introduction to add official demographic context for the broader national youth category that includes the study’s target age range. Specifically, I added national youth statistics from the General Authority for Statistics indicating that Saudi youth are officially defined as individuals aged (15–34), representing (36.2%) of the Saudi population, while Saudis under the age of (35) represent approximately (71%) of the total Saudi population. I then clarified that the present study focused on the narrower age range of (18–25) because it represents a more developmentally coherent subgroup corresponding to emerging adulthood and reduces age-related heterogeneity in psychometric validation.

**Comment 1.3 – "The Present research" section**

*Include a section titled "The present research," specifying the objectives and, especially, the hypotheses in order to improve the organization of the study.*

**Response:**

Thank you for this helpful suggestion. I added a subsection titled "The Present research" at the end of the Introduction to improve the organization of the study and to make the objectives and hypotheses explicit. The new subsection now states the study’s three objectives: examining the factor structure of the Arabic PDWS using exploratory and confirmatory factor analyses, including the second-order hierarchical specification; evaluating reliability using Cronbach’s alpha and McDonald’s omega; and assessing convergent validity through associations with Fear of Missing Out (FoMO) and life satisfaction. I also added three corresponding hypotheses concerning the expected three-domain and higher-order structure of the Arabic PDWS, acceptable internal consistency reliability across domains and for the total score, and theoretically consistent associations with life satisfaction and FoMO.

**Comment 2.1 – Nature of the Sample**

*Specify the nature of the sample: non-probabilistic?*

**Response:**

Thank you for this clarification request. I revised the Participants subsection to explicitly describe the nature of the sample. The manuscript now states that participants were recruited using a non-probabilistic convenience sampling approach through online dissemination of the survey link on social media platforms in Saudi Arabia. I also clarified that the full sample was then randomly split into two equal analytic subsamples for the EFA and CFA procedures. This clarification distinguishes the recruitment strategy from probability-based sampling and makes the sampling approach more transparent.

**Comment 2.2 – Social Media Platforms Used for Recruitment**

*How was data collection disseminated? Through which specific social media platforms or networks?*

**Response:**

Thank you for this clarification request. I revised the Procedure subsection to specify the social media platforms used for recruitment. The manuscript now states that participants were recruited through WhatsApp, Snapchat, X (formerly Twitter), and Instagram in Saudi Arabia between 15 March 2025 and 30 May 2025.

**Comment 2.3 – Justification for Split-Sample Design**

*Justify the procedure of splitting the sample into two and testing it across different samples (exploratory and confirmatory analyses).*

**Response:**

Thank you for this helpful methodological comment. I revised the Study Design and setting subsection to justify the split-sample procedure. The manuscript now explains that the sample was split to provide a more rigorous cross-validation of the factor structure: EFA was conducted in one subsample to identify the underlying structure, whereas CFA was conducted in an independent subsample to test the replicability and fit of the resulting measurement model. This approach reduces the risk of capitalizing on sample-specific variation when exploratory and confirmatory analyses are performed on the same participants.

**Comment 2.4 – Justification of Sample Sizes**

*Justify the sample sizes for both multivariate analytical procedures.*

**Response:**

Thank you for this comment. I revised the Participants subsection to justify the sample sizes used for the two multivariate analytical procedures. The manuscript now states that the EFA subsample (n=225) provided approximately (13.2) participants per item for the (17)-item PDWS, exceeding the commonly used (10:1) participant-to-item heuristic. It also clarifies that the CFA subsample (n=225) was considered sufficient for testing a (17)-item measurement model with three first-order domains and a second-order factor, taking into account that CFA/SEM sample size requirements depend on model complexity, number of indicators, factor loadings, and related design features, as discussed by Wolf et al. (2013).

**Comment 2.5 – Sociodemographic Table**

*Include a table presenting the sociodemographic characteristics of Samples 1 and 2, as well as the total sample.*

**Response:**

A sociodemographic characteristics table has been added to the Participants subsection, presenting Sample 1 (EFA subsample; n=225), Sample 2 (CFA subsample; n=225), and the total sample (N=450). The table reports age (M, SD, range), gender, marital status, and education, and it is explicitly cited in the Participants text (Table 1).

**Comment 2.6 – Second-Order vs. Bifactor Model**

*Justify why a second-order hierarchical model was also tested instead of a bifactor model.*

**Response:**

Thank you for this important methodological question. I revised the Methods section to justify why a second-order hierarchical model was specified rather than a bifactor model. The manuscript now explains that the PDWS was theoretically designed as a hierarchical construct in which Emotional, Social, and Cognitive domain-specific appraisals reflect an overarching perceived digital well-being factor. I also clarified that the expected intercorrelations among the three domains are more directly represented by a second-order structure, whereas a bifactor model would partition item variance into a general factor and residual specific factors. Finally, I added that second-order and bifactor models differ in the interpretation of the general factor; the second-order model was therefore considered more conceptually consistent and parsimonious for the aims of this initial Arabic validation.

**Comment 2.7 – CFA Results in Instrument Section**

*In the instrument section, include the results of the confirmatory analysis based on the samples from the present study rather than from previous studies.*

**Response:**

Thank you for this clarification. I added a sentence to the PDWS description in the Measures subsection to explicitly reference the psychometric evidence from the present study’s own CFA, rather than relying only on prior validation studies. Specifically, the revised text now notes that CFA in the independent CFA subsample supported a well-fitting second-order model, with all standardized factor loadings statistically significant, and directs readers to the Results section for full details. This makes the instrument description clearer with respect to the current study’s data.

**Comment 2.8 – Extraction Method in EFA**

*Which extraction method was used: Kaiser-Guttman Criterion, Scree Plot, parallel analysis? Explicitly state this aspect.*

**Response:**

Thank you for this clarification request. I revised the EFA Results subsection to distinguish more explicitly between the extraction method and the factor-retention criteria. The manuscript now states that maximum likelihood extraction was used with Direct Oblimin rotation and Kaiser normalization. It also clarifies that factor retention was guided by the Kaiser-Guttman criterion (eigenvalues>1), visual inspection of the scree plot, and the maximum-likelihood goodness-of-fit test for the retained solution. The retained three-factor solution was further supported by the nonsignificant ML goodness-of-fit test, χ²(88)=82.597, p=.643, indicating adequate fit of the three-factor solution.

**Comment 2.9 – Justification for Maximum Likelihood in CFA**

*For the confirmatory factor analysis, justify the use of the maximum likelihood (ML) method. Describe the normality of the data to assess whether this was the most appropriate method, considering the categorical-ordinal nature of the instrument.*

**Response:**

Thank you for this important methodological comment. I revised the CFA-related Methods text to justify the use of maximum likelihood estimation and to address the ordinal nature of the five-point response format. Specifically, I inspected item-level distributions in the CFA subsample before model estimation. All items had complete data (N=225; no missing values). Skewness values ranged from (-0.860) to (0.078), and kurtosis values ranged from (-0.796) to (0.198), indicating no severe univariate non-normality. Given the five-point response format, acceptable item-level distributional properties, and evidence that ordinal variables with five or more response categories can often be treated as approximately continuous in SEM under non-severe non-normality, maximum likelihood estimation was considered appropriate for testing the second-order CFA model in the present sample.

**Comment 2.10 – Measurement Invariance**

*Given that confirmatory factor analysis was conducted, it is important to provide evidence of measurement invariance (i.e., configural, metric, scalar, and strict invariance) across certain sociodemographic variables (e.g., gender, education), since the authors have these variables in their dataset.*

**Response:**

Thank you for this important suggestion. I conducted multi-group CFA in the CFA subsample (n=225) to test measurement invariance across (gender: male vs female) and (education: high school or less vs bachelor vs postgraduate), following the standard stepwise sequence of configural, metric (equal factor loadings), scalar (equal intercepts), and strict (equal residuals) invariance.

Across gender, model fit remained stable across increasingly constrained models (configural: CFI=0.954, RMSEA=0.033; metric: CFI=0.954, RMSEA=0.032; scalar: CFI=0.947, RMSEA=0.034; strict: CFI=0.945, RMSEA=0.033), with small changes in fit indices (maximum ΔCFI=0.007; ΔRMSEA=0.002), supporting scalar and strict invariance.

Across education level (three groups), invariance was also supported (configural: CFI=0.938, RMSEA=0.032; metric: CFI=0.928, RMSEA=0.033; scalar: CFI=0.936, RMSEA=0.030; strict: CFI=0.939, RMSEA=0.028). The metric step showed a borderline but acceptable change (ΔCFI=0.010; ΔRMSEA=0.001), and subsequent steps remained within recommended thresholds, supporting scalar and strict invariance across education level.

These results were added to the Methods section and summarized in the Results section (Tables 10–11).

**Comment 3.1 – Figure Quality**

*Provide figures in higher quality (e.g., TIFF format).*

**Response:**

Thank you for this comment. I re-exported Fig 1 in high-resolution TIFF format to improve readability and production quality. The revised figure file will be uploaded separately as Fig1.tif in accordance with the journal’s figure-quality requirements.

**Comment 4.1 – Practical implications**

*Include a section on practical implications contextualized to the country of origin of the sample.*

**Response:**

Thank you for this valuable suggestion. I added a new subsection titled "Practical implications" to the Discussion before the Limitations and Future Directions section. The new subsection contextualizes the applied relevance of the Arabic PDWS within Saudi Arabia, with specific implications for research on Saudi young adults, university counseling and student well-being services, psychoeducational programs, and institutional initiatives aimed at promoting healthier digital engagement. I also emphasized that the multidimensional structure of the PDWS can help distinguish emotional, social, and cognitive aspects of perceived digital well-being, supporting more targeted assessment and intervention planning.

**Comment 5.1 – Artificial intelligence statement**

*Include an Artificial intelligence statement specifying whether it was used, and if so, in which sections and for what purposes.*

**Response:**

Thank you for this important request. I added an Artificial intelligence statement after the Conflicts of Interest Statement. The statement discloses that ChatGPT (OpenAI) was used during manuscript revision to support language editing, organization of reviewer responses, and refinement of selected manuscript text under the author’s supervision. It also clarifies that AI was not used to generate data, conduct statistical analyses, create or alter scientific figures, or determine the study findings. I reviewed, verified, and approved all AI-assisted text and remain fully responsible for the accuracy, integrity, citations, analyses, and conclusions of the manuscript.

**Reviewer 2 Comments**

**Comment – Smartphone and Internet Use Statistics in Saudi Arabia**

*Introduction: After the first paragraph, it would be important to include data that supports the statements made. Please add information on smartphone and internet use in the country. In the ranking of social media users, what position does the country hold globally?*

**Response:**

Thank you for this helpful suggestion. I revised the Introduction by adding a data-supported paragraph after the opening paragraph to contextualize digital technology use in Saudi Arabia. The new paragraph reports recent national and international indicators, including internet penetration in Saudi Arabia, mobile phone use for internet browsing, the number of internet users and cellular mobile connections, and the number of social media user identities. Because I did not identify a reliable source providing a single global ranking position for Saudi Arabia in overall social media use, I avoided reporting an unsupported rank. Instead, I contextualized Saudi Arabia’s social media penetration by noting that social media user identities represented (99.6%) of the total population in January (2025), substantially above the global social media user-identity penetration of (63.9%). This provides a clear and evidence-based basis for describing Saudi Arabia as a highly connected digital context.

**Comment – Justification for Study Population**

*A more robust justification for the study population is needed, given that sample diversity is a fundamental requirement for the validation of an instrument.*

**Response:**

Thank you for this important comment. I strengthened the justification for the study population in the Introduction and clarified the sampling scope and generalizability limits in the Methods and Limitations sections. The revised Introduction now explains that the study focused on Saudi young adults aged (18–25) because this age range corresponds to emerging adulthood, a developmental period in which identity exploration, peer relationships, and digitally mediated routines are especially salient. I also added official Saudi youth statistics to contextualize this target population within the broader national youth category. Methodologically, the focus on a narrower age range was intended to reduce age-related heterogeneity that could obscure factor structure and validity evidence in an initial validation study. At the same time, I explicitly acknowledged in the Limitations section that the sample was recruited online, was limited to Saudi young adults in Riyadh, and that generalization to other regions, age groups, or Arabic-speaking populations should be made cautiously. Future research with more diverse and representative samples is recommended to extend the present validation evidence.

**Comment – Flow Issue at Line 71 (Transition Paragraph)**

*The first paragraph on page 4 (line 71) does not connect well with the preceding one, resulting in a sudden and abrupt break in the flow of ideas.*

**Response:**

Thank you for this helpful observation. I revised the transition into the PDWS paragraph in the Introduction to improve the flow of ideas. Specifically, I added a linking sentence that connects the preceding conceptual, cultural, and developmental rationale to the need for a measure that captures how Saudi young adults themselves appraise the perceived impact of digital engagement on everyday functioning. This revision makes the transition to the PDWS more explicit and reduces the abrupt shift noted by the reviewer.

**Comment – Life Satisfaction and Online Experiences**

*Regarding life satisfaction, it is necessary to clarify more clearly how it relates to online experiences. Additionally, it should be made explicit whether satisfaction is also intended to be conceptualized in relation to the online environment.*

**Response:**

Thank you for this clarifying comment. I revised the Introduction to make the role of life satisfaction clearer. The manuscript now explicitly states that life satisfaction was not conceptualized as satisfaction with online experiences specifically; rather, it was treated as a domain-general indicator of evaluative well-being and as a distal convergent criterion. I also clarified the theoretical rationale: if perceived digital well-being captures how smartphone use supports emotional, social, and cognitive functioning in daily life, it should show a positive association with broader evaluations of life quality. This clarification distinguishes SWLS from digital-specific satisfaction while explaining why it is theoretically appropriate as a convergent validity benchmark.

**Comment – Item Response Theory (IRT) or SEM**

*Methods: I suggest the use of Item Response Theory (IRT) or Structural Equation Modeling (SEM).*

**Response:**

Thank you for this suggestion. I clarified that the present study already used structural equation modeling (SEM) through confirmatory factor analysis (CFA) and multi-group CFA for measurement invariance testing in AMOS. These analyses provided SEM-based evidence for the factor structure and measurement equivalence of the Arabic PDWS. Regarding item response theory (IRT), I agree that IRT models could provide valuable complementary item-level information, such as discrimination, threshold parameters, and response category functioning. However, IRT analysis was beyond the scope of the present initial validation study, which focused on classical and SEM-based psychometric evidence. I added this point to the Limitations and Future Directions section as a recommendation for future research.

**Comment – Weak to Moderate Correlations**

*Results: Although the study reports correlations, only a few were strong, while the majority ranged from weak to moderate. This pattern should be further discussed.*

**Response:**

Thank you for this important observation. I added a discussion of the magnitude of the convergent validity correlations in the Discussion section. The revised text now acknowledges that most associations were small to moderate rather than strong and interprets this pattern in relation to the conceptual distance between the PDWS and the two convergent benchmarks. I clarified that SWLS and FoMO were selected as distal convergent criteria, not as measures of the same construct. I also noted that small average associations are common in the broader digital technology and well-being literature. Therefore, the convergent validity evidence is interpreted primarily in terms of the consistent direction and replication of the associations across the two independent subsamples—positive associations with life satisfaction and negative associations with FoMO—rather than expecting uniformly strong correlations.

**Comment – Item Saturation and Underlying Phenomenon**

*Overall, although the instrument appears statistically adequate, the analyses could be expanded further. It would be valuable to explore item saturation and the underlying phenomenon in greater depth. More robust analytical approaches would allow for a more comprehensive understanding of the construct and its psychosocial relationships.*

**Response:**

Thank you for this valuable suggestion. I expanded the Discussion to address item saturation and the underlying phenomenon in greater depth. The revised text now interprets the standardized factor loadings and squared multiple correlations (R²/SMC) reported in Table 5 as item-level evidence of saturation. I clarified that Cognitive items showed the strongest and most consistent explained variance, Social items showed moderate-to-strong values, and Emotional items showed more variable saturation, suggesting that perceived digital well-being is a multidimensional and context-sensitive appraisal of smartphone-related functioning rather than a single undifferentiated construct. I also expanded the Limitations and Future Directions section to recommend future use of item response theory and network psychometric approaches to examine item-level discrimination, threshold parameters, response category functioning, and item-to-item relationships.
