# Supplementary material for: Psychometric properties of the Arabic Perceived Digital Well-Being Scale (PDWS) in Saudi young adults
Source: PLoS One. 2026 Jul 30;21(7):e0354474. doi: 10.1371/journal.pone.0354474 (PMC13422880; doi:10.1371/journal.pone.0354474)
Supplement: Attachment — Submitted filename: PDWS_Response_to_Reviewers.docx [file pone.0354474.s005.docx]

**Response to Reviewers**

**Manuscript ID: PONE-D-26-11267R1**

Psychometric properties of the Arabic Perceived Digital Well-Being Scale (PDWS) in Saudi young adults

PLOS ONE

**Dear Dr. Mariano and Reviewers,**

I thank the Academic Editor and the reviewers for their careful and constructive evaluation of my manuscript. I am pleased that the reviewers regarded the work as technically sound, the statistical analyses as appropriate, the data as fully available, and the manuscript as clearly written. The manuscript has been revised to address every point raised. Below I respond to each comment in turn; the reviewer’s comment is shown first, followed by the response. All changes are highlighted in the "Revised Manuscript with Track Changes" file. To avoid any mismatch across versions, I refer to the relevant section of the manuscript (e.g., Results, Discussion) rather than to specific page or line numbers. No new analyses were conducted; all reported values remain unchanged and were already included in the manuscript’s tables. The reference list was reviewed for completeness and accuracy, and no changes were required.

**Reviewer #3**

***Comment.*** *The article addresses a highly relevant topic and is methodologically sophisticated, with a coherent split-sample design and adequate translation and ethics procedures. However, an analysis of the construct reliability and validity table reveals a psychometric issue that should be addressed: under the Fornell–Larcker criterion, the square root of the AVE for the Emotional dimension (0.571) is lower than its correlations with the Social (r = 0.669) and Cognitive (r = 0.678) dimensions, and the square root of the AVE for the Social dimension (0.667) is also slightly lower than its correlation with the Emotional dimension (r = 0.669). The factors thus share more variance with one another than with their own indicators. A plausible justification is that the second-order specification (a general Digital Well-Being factor) absorbs this high latent correlation. The author should critically report and discuss the AVE values and the violation of discriminant validity between the Emotional and Social factors in Sample 2.*

**Response.** I thank the reviewer for this careful and constructive observation, and I agree that the original manuscript did not report or discuss these results with sufficient transparency. I have addressed this in two places, using the values already presented in Table 9 (no values were changed and no new analyses were performed). First, in the Results (Construct reliability and validity), I now explicitly report that the AVE was below the conventional .50 threshold for the Emotional (AVE = .326) and Social (AVE = .445) dimensions and above it for the Cognitive dimension (AVE = .593), and I state that the square root of the AVE for the Emotional dimension (.571) was lower than its correlations with the Social (.669) and Cognitive (.678) dimensions, while the square root of the AVE for the Social dimension (.667) was marginally lower than its correlation with the Emotional dimension (.669); the Cognitive dimension met the criterion. I therefore state plainly that the Fornell–Larcker discriminant validity criterion was not satisfied for the Emotional and Social dimensions. Second, in the Discussion, I now interpret this pattern critically and adopt the explanation the reviewer helpfully suggested: the elevated first-order inter-domain correlations are theoretically coherent with the second-order specification, in which the three domains function as strong reflective indicators of an overarching factor (second-order loadings: Emotional = .894, Social = .749, Cognitive = .759; Table 6), so that high inter-domain correlations are expected and mechanically constrain first-order discriminant validity as indexed by Fornell–Larcker. I also acknowledge this issue in the Limitations and recommend that future work examine first-order discriminant validity using complementary discriminant-validity indices and larger, more diverse samples. I am grateful to the reviewer, as these revisions have improved the transparency and rigor of the manuscript.

**Reviewer #4**

The manuscript presents adequate data and interpretations to corroborate its findings and discusses a relevant topic. I thank the reviewer for the positive assessment and for two helpful suggestions.

**Comment 1.**

***Comment.*** *In the description of the scales, add an item representing each factor just as an example.*

**Response.** I thank the reviewer for this suggestion. In the Measures section (PDWS description), I have added a representative bipolar example item for each of the three domains and clarified the response anchors (1 = negative pole; 5 = positive pole): Emotional (E2)—"Because of my smartphone use, I feel more stressed" versus "… I feel more relaxed"; Social (S8)—"… I feel more excluded from my friends" versus "… I feel closer to my friends"; Cognitive (C14)—"… I do less of my daily tasks (e.g., schoolwork, university work, and professional work)" versus "… I do more of my daily tasks". I also refer readers to S1 and S2 Appendices, which contain the complete Arabic and English item sets.

**Comment 2.**

***Comment.*** *Why was the maximum likelihood (ML) estimator used? For this sample size, ULS and DWLS were more suited.*

**Response.** I appreciate this methodological point and have clarified the rationale in the Confirmatory factor analysis model specification subsection. ML estimation was selected because the PDWS items use a five-point response format and the item-level distributions in the CFA subsample were approximately normal (skewness ranged from −0.860 to 0.078 and kurtosis from −0.796 to 0.198, with no severe univariate non-normality). Under these conditions—at least five ordered categories combined with approximately symmetric, near-normal distributions—established simulation evidence shows that treating the items as continuous and applying ML yields parameter estimates, standard errors, and fit indices that are comparable to those obtained from categorical estimators such as ULS/DWLS, whose relative advantages are most pronounced with fewer response categories and pronounced non-normality (Rhemtulla, Brosseau-Liard, & Savalei, 2012; reference [29]). Consistent with this, the ML solution produced excellent fit (e.g., CFI = .986, RMSEA = .026, SRMR = .055). I have added this justification to the manuscript.

These revisions address the reviewers’ comments and strengthen the transparency and methodological reporting of the manuscript. I thank the Editor and reviewers for their time and valuable feedback.

Sincerely,

**Emadeldin M. Elsokkary**

Department of Psychology, College of Social Sciences

Imam Mohammad Ibn Saud Islamic University (IMSIU), Riyadh, Saudi Arabia

Email: [emelsokkary@imamu.edu.sa](mailto:emelsokkary@imamu.edu.sa)
