# Supplementary material for: Psychometric properties of the Arabic Perceived Digital Well-Being Scale (PDWS) in Saudi young adults
Source: PLoS One. 2026 Jul 30;21(7):e0354474. doi: 10.1371/journal.pone.0354474 (PMC13422880; doi:10.1371/journal.pone.0354474)
Supplement: Peer review history

# Peer review: Decision Letter 0

**DOI:** 10.1371/journal.pone.0354474.r001

**Authors:** Tailson Mariano

27 Apr 2026

PONE-D-26-11267

Psychometric Properties of the Arabic Perceived Digital Well-Being Scale (PDWS) in Saudi Young Adults

PLOS One

Dear Dr.Elsokkary,

Thank you for submitting your manuscript to PLOS ONE. After careful consideration, we feel that it has merit but does not fully meet PLOS ONE’s publication criteria as it currently stands. Therefore, we invite you to submit a revised version of the manuscript that addresses the points raised during the review process.

Please submit your revised manuscript by Jun 11 2026 11:59PM. If you will need more time than this to complete your revisions, please reply to this message or contact the journal office at plosone@plos.org. When you're ready to submit your revision, log on to https://www.editorialmanager.com/pone/ and select the 'Submissions Needing Revision' folder to locate your manuscript file.

Please include the following items when submitting your revised manuscript:

- A letter that responds to each point raised by the academic editor and reviewer(s). You should upload this letter as a separate file labeled 'Response to Reviewers'.
- A marked-up copy of your manuscript that highlights changes made to the original version. You should upload this as a separate file labeled 'Revised Manuscript with Track Changes'.
- An unmarked version of your revised paper without tracked changes. You should upload this as a separate file labeled 'Manuscript'.

If you would like to make changes to your financial disclosure, please include your updated statement in your cover letter. Guidelines for resubmitting your figure files are available below the reviewer comments at the end of this letter.

If applicable, we recommend that you deposit your laboratory protocols in protocols.io to enhance the reproducibility of your results. Protocols.io assigns your protocol its own identifier (DOI) so that it can be cited independently in the future. For instructions see: https://journals.plos.org/plosone/s/submission-guidelines#loc-laboratory-protocols. Additionally, PLOS ONE offers an option for publishing peer-reviewed Lab Protocol articles, which describe protocols hosted on protocols.io. Read more information on sharing protocols at https://plos.org/protocols?utm_medium=editorial-email&utm_source=authorletters&utm_campaign=protocols.

As the corresponding author, your ORCID iD is verified in the submission system and will appear in the published article. PLOS supports the use of ORCID, and we encourage all coauthors to register for an ORCID iD and use it as well. Please encourage your coauthors to verify their ORCID iD within the submission system before final acceptance, as unverified ORCID iDs will not appear in the published article. *Only*  the individual author can complete the verification step; PLOS staff *cannot*  verify ORCID iDs on behalf of authors.

We look forward to receiving your revised manuscript.

Kind regards,

Tailson Evangelista Mariano, Ph.D.

Academic Editor

PLOS One

Journal Requirements:

When submitting your revision, we need you to address these additional requirements.

1.Please ensure that your manuscript meets PLOS ONE's style requirements, including those for file naming. The PLOS ONE style templates can be found at

https://journals.plos.org/plosone/s/file?id=wjVg/PLOSOne_formatting_sample_main_body.pdf and

https://journals.plos.org/plosone/s/file?id=ba62/PLOSOne_formatting_sample_title_authors_affiliations.pdf

2. Thank you for stating the following financial disclosure:

“This work was supported and funded by the Deanship of Scientific Research at Imam Mohammad Ibn Saud Islamic University (IMSIU) (grant number IMSIU-DDRSP2601).”

Please state what role the funders took in the study.  If the funders had no role, please state: "The funders had no role in study design, data collection and analysis, decision to publish, or preparation of the manuscript."

If this statement is not correct you must amend it as needed.

Please include this amended Role of Funder statement in your cover letter; we will change the online submission form on your behalf.

3. Please note that your Data Availability Statement is currently missing the repository name and/or the DOI/accession number of each dataset OR a direct link to access each database. If your manuscript is accepted for publication, you will be asked to provide these details on a very short timeline. We therefore suggest that you provide this information now, though we will not hold up the peer review process if you are unable.

4. Please ensure that you refer to Figure 1 in your text as, if accepted, production will need this reference to link the reader to the figure.

5. Please remove your figures from within your manuscript file, leaving only the individual TIFF/EPS image files, uploaded separately. These will be automatically included in the reviewers’ PDF.

6. We note you have included a table to which you do not refer in the text of your manuscript. Please ensure that you refer to Table 1-7 in your text; if accepted, production will need this reference to link the reader to the Table.

7. If the reviewer comments include a recommendation to cite specific previously published works, please review and evaluate these publications to determine whether they are relevant and should be cited. There is no requirement to cite these works unless the editor has indicated otherwise.

[Note: HTML markup is below. Please do not edit.]

Reviewers' comments:

Reviewer's Responses to Questions

**Comments to the Author**

1. Is the manuscript technically sound, and do the data support the conclusions?

The manuscript must describe a technically sound piece of scientific research with data that supports the conclusions. Experiments must have been conducted rigorously, with appropriate controls, replication, and sample sizes. The conclusions must be drawn appropriately based on the data presented.

Reviewer #1: Yes

Reviewer #2: Partly

**********

2. Has the statistical analysis been performed appropriately and rigorously?

Reviewer #1: Yes

Reviewer #2: Yes

**********

3. Have the authors made all data underlying the findings in their manuscript fully available?

The PLOS Data policy requires authors to make all data underlying the findings described in their manuscript fully available without restriction, with rare exception (please refer to the Data Availability Statement in the manuscript PDF file). The data should be provided as part of the manuscript or its supporting information, or deposited to a public repository. For example, in addition to summary statistics, the data points behind means, medians and variance measures should be available. If there are restrictions on publicly sharing data—e.g. participant privacy or use of data from a third party—those must be specified.

Reviewer #1: Yes

Reviewer #2: Yes

**********

4. Is the manuscript presented in an intelligible fashion and written in standard English?

PLOS ONE does not copyedit accepted manuscripts, so the language in submitted articles must be clear, correct, and unambiguous. Any typographical or grammatical errors should be corrected at revision, so please note any specific errors here.

Reviewer #1: Yes

Reviewer #2: Yes

**********

5. Review Comments to the Author

Please use the space provided to explain your answers to the questions above. You may also include additional comments for the author, including concerns about dual publication, research ethics, or publication ethics. (Please upload your review as an attachment if it exceeds 20,000 characters)

Reviewer #1: Review Comments to the Author - Psychometric Properties of the Arabic Perceived Digital Well-Being Scale (PDWS) in Saudi Young Adults

The manuscript addresses a relevant topic and presents preliminary data on the Perceived Digital Well-Being Scale in a context where such data are scarce. Considering these aspects, I outline below the main suggestions aimed at achieving a version suitable for publication.

1. Introduction

1.1. Explicitly state the origin of the PDWS instrument and its previous evidence, including content validity, internal and external structure, and adaptations in other contexts, among others.

1.2. Include demographic statistics (i.e., National Population Census) regarding the age range of the study’s target population in this country.

1.3. Include a section titled “The present research,” specifying the objectives and, especially, the hypotheses in order to improve the organization of the study.

2. Methods

2.1. Specify the nature of the sample: non-probabilistic?

2.2. How was data collection disseminated? Through which specific social media platforms or networks?

2.3. Justify the procedure of splitting the sample into two and testing it across different samples (exploratory and confirmatory analyses).

2.4. Justify the sample sizes for both multivariate analytical procedures.

2.5. Include a table presenting the sociodemographic characteristics of Samples 1 and 2, as well as the total sample.

2.6. Justify why a second-order hierarchical model was also tested instead of a bifactor model.

2.7. In the instrument section, include the results of the confirmatory analysis based on the samples from the present study rather than from previous studies.

2.8. Which extraction method was used: Kaiser-Guttman Criterion, Scree Plot, parallel analysis? Explicitly state this aspect.

2.9. For the confirmatory factor analysis, justify the use of the maximum likelihood (ML) method. Describe the normality of the data to assess whether this was the most appropriate method, considering the categorical-ordinal nature of the instrument.

2.10. Given that confirmatory factor analysis was conducted, it is important to provide evidence of measurement invariance (i.e., configural, metric, scalar, and strict invariance) across certain sociodemographic variables (e.g., gender, education), since the authors have these variables in their dataset.

3. Results

3.1. Provide figures in higher quality (e.g., TIFF format).

4. Discussion

4.1. Include a section on practical implications contextualized to the country of origin of the sample.

5. Additional Suggestions

5.1. Include an Artificial Intelligence statement specifying whether it was used, and if so, in which sections and for what purposes.

Reviewer #2: The manuscript presents a relevant contribution to the field of study, addressing a current and potentially significant topic for the scientific literature. The overall structure of the work is appropriate, with well-organized sections and a logical presentation of the objectives, methods, results, and conclusions. However, it requires revisions that would enrich the work:

Introduction: After the first paragraph, it would be important to include data that supports the statements made. Please add information on smartphone and internet use in the country. In the ranking of social media users, what position does the country hold globally?

A more robust justification for the study population is needed, given that sample diversity is a fundamental requirement for the validation of an instrument.

The first paragraph on page 4 (line 71) does not connect well with the preceding one, resulting in a sudden and abrupt break in the flow of ideas.

Regarding life satisfaction, it is necessary to clarify more clearly how it relates to online experiences. Additionally, it should be made explicit whether satisfaction is also intended to be conceptualized in relation to the online environment.

Methods: I suggest the use of Item Response Theory (IRT) or Structural Equation Modeling (SEM).

Results: Although the study reports correlations, only a few were strong, while the majority ranged from weak to moderate. This pattern should be further discussed.

Overall, although the instrument appears statistically adequate, the analyses could be expanded further. It would be valuable to explore item saturation and the underlying phenomenon in greater depth. More robust analytical approaches would allow for a more comprehensive understanding of the construct and its psychosocial relationships.

**********

6. PLOS authors have the option to publish the peer review history of their article (what does this mean?). If published, this will include your full peer review and any attached files.

If you choose “no”, your identity will remain anonymous but your review may still be made public.

**Do you want your identity to be public for this peer review?**  For information about this choice, including consent withdrawal, please see our Privacy Policy.

Reviewer #1: No

Reviewer #2: No

**********

[NOTE: If reviewer comments were submitted as an attachment file, they will be attached to this email and accessible via the submission site. Please log into your account, locate the manuscript record, and check for the action link "View Attachments". If this link does not appear, there are no attachment files.]

To ensure your figures meet our technical requirements, please review our figure guidelines: https://journals.plos.org/plosone/s/figures

You may also use PLOS’s free figure tool, NAAS, to help you prepare publication quality figures: https://journals.plos.org/plosone/s/figures#loc-tools-for-figure-preparation.

NAAS will assess whether your figures meet our technical requirements by comparing each figure against our figure specifications.

# Author comment: Author response to Decision Letter 1

**DOI:** 10.1371/journal.pone.0354474.r002

14 May 2026

Dear Editor,

A detailed point-by-point response to the journal requirements and reviewer comments has been uploaded as “Response_to_Reviewers_PONE-D-26-11267.docx”. The revised clean manuscript, the marked-up manuscript with all changes highlighted in red, the updated Fig1.tif file, and the supporting information files have also been uploaded.

Kind regards,

Emadeldin M. Elsokkary

Attachment Submitted filename: Response_to_Reviewers_PONE-D-26-11267.docx

# Peer review: Decision Letter 1

**DOI:** 10.1371/journal.pone.0354474.r003

**Authors:** Tailson Mariano

17 Jun 2026

PONE-D-26-11267R1

Psychometric properties of the Arabic Perceived Digital Well-Being Scale (PDWS) in Saudi young adults

PLOS One

Dear Dr. Elsokkary,

Thank you for submitting your manuscript to PLOS ONE. After careful consideration, we feel that it has merit but does not fully meet PLOS ONE’s publication criteria as it currently stands. Therefore, we invite you to submit a revised version of the manuscript that addresses the points raised during the review process.

Please submit your revised manuscript by Aug 01 2026 11:59PM. If you will need more time than this to complete your revisions, please reply to this message or contact the journal office at plosone@plos.org. When you're ready to submit your revision, log on to https://www.editorialmanager.com/pone/ and select the 'Submissions Needing Revision' folder to locate your manuscript file.

Please include the following items when submitting your revised manuscript:

- A letter that responds to each point raised by the academic editor and reviewer(s). You should upload this letter as a separate file labeled 'Response to Reviewers'.
- A marked-up copy of your manuscript that highlights changes made to the original version. You should upload this as a separate file labeled 'Revised Manuscript with Track Changes'.
- An unmarked version of your revised paper without tracked changes. You should upload this as a separate file labeled 'Manuscript'.

If you would like to make changes to your financial disclosure, please include your updated statement in your cover letter. Guidelines for resubmitting your figure files are available below the reviewer comments at the end of this letter.

If applicable, we recommend that you deposit your laboratory protocols in protocols.io to enhance the reproducibility of your results. Protocols.io assigns your protocol its own identifier (DOI) so that it can be cited independently in the future. For instructions see: https://journals.plos.org/plosone/s/submission-guidelines#loc-laboratory-protocols. Additionally, PLOS ONE offers an option for publishing peer-reviewed Lab Protocol articles, which describe protocols hosted on protocols.io. Read more information on sharing protocols at https://plos.org/protocols?utm_medium=editorial-email&utm_source=authorletters&utm_campaign=protocols.

As the corresponding author, your ORCID iD is verified in the submission system and will appear in the published article. PLOS supports the use of ORCID, and we encourage all coauthors to register for an ORCID iD and use it as well. Please encourage your coauthors to verify their ORCID iD within the submission system before final acceptance, as unverified ORCID iDs will not appear in the published article. *Only*  the individual author can complete the verification step; PLOS staff *cannot*  verify ORCID iDs on behalf of authors.

We look forward to receiving your revised manuscript.

Kind regards,

Tailson Evangelista Mariano, Ph.D.

Academic Editor

PLOS One

Journal Requirements:

1. If the reviewer comments include a recommendation to cite specific previously published works, please review and evaluate these publications to determine whether they are relevant and should be cited. There is no requirement to cite these works unless the editor has indicated otherwise.

2. Please review your reference list to ensure that it is complete and correct. If you have cited papers that have been retracted, please include the rationale for doing so in the manuscript text, or remove these references and replace them with relevant current references. Any changes to the reference list should be mentioned in the rebuttal letter that accompanies your revised manuscript. If you need to cite a retracted article, indicate the article’s retracted status in the References list and also include a citation and full reference for the retraction notice.

[Note: HTML markup is below. Please do not edit.]

Reviewers' comments:

Reviewer's Responses to Questions

**Comments to the Author**

1. If the authors have adequately addressed your comments raised in a previous round of review and you feel that this manuscript is now acceptable for publication, you may indicate that here to bypass the “Comments to the Author” section, enter your conflict of interest statement in the “Confidential to Editor” section, and submit your "Accept" recommendation.

Reviewer #3: (No Response)

Reviewer #4: (No Response)

**********

2. Is the manuscript technically sound, and do the data support the conclusions?

The manuscript must describe a technically sound piece of scientific research with data that supports the conclusions. Experiments must have been conducted rigorously, with appropriate controls, replication, and sample sizes. The conclusions must be drawn appropriately based on the data presented.

Reviewer #3: Yes

Reviewer #4: Yes

**********

3. Has the statistical analysis been performed appropriately and rigorously?

Reviewer #3: Yes

Reviewer #4: Yes

**********

4. Have the authors made all data underlying the findings in their manuscript fully available?

The PLOS Data policy requires authors to make all data underlying the findings described in their manuscript fully available without restriction, with rare exception (please refer to the Data Availability Statement in the manuscript PDF file). The data should be provided as part of the manuscript or its supporting information, or deposited to a public repository. For example, in addition to summary statistics, the data points behind means, medians and variance measures should be available. If there are restrictions on publicly sharing data—e.g. participant privacy or use of data from a third party—those must be specified.

Reviewer #3: Yes

Reviewer #4: Yes

**********

5. Is the manuscript presented in an intelligible fashion and written in standard English?

PLOS ONE does not copyedit accepted manuscripts, so the language in submitted articles must be clear, correct, and unambiguous. Any typographical or grammatical errors should be corrected at revision, so please note any specific errors here.

Reviewer #3: Yes

Reviewer #4: Yes

**********

6. Review Comments to the Author

Please use the space provided to explain your answers to the questions above. You may also include additional comments for the author, including concerns about dual publication, research ethics, or publication ethics. (Please upload your review as an attachment if it exceeds 20,000 characters)

Reviewer #3: The article under evaluation addresses a theme of high contemporary relevance and growing expansion within Media Psychology and Psychometrics: digital well-being. The author performs the translation, cultural adaptation, and validation of the Perceived Digital Well-Being Scale (PDWS) into the Arabic language, utilizing a sample of 450 young adults from Saudi Arabia.The manuscript's primary theoretical virtue lies in moving away from the traditional pathologizing approach in the literature (which focuses almost exclusively on "smartphone addiction" or "problematic screen use") to adopt a subject-centered perspective focused on the individual's subjective assessment of technology's impact on their spheres of functioning. From a psychometric standpoint, the study is sophisticated and adopts a coherent methodological split-sample design, executing exploratory and confirmatory analyses on independent databases.Technical Standard of Experiments, Statistical Analyses, and Methodological DetailsThe collection methodology, translation procedures, and sample size ($N = 450$, split into two subsamples of $n = 225$) are technically adequate. However, an analysis of Table 8 reveals psychometric inconsistencies that were not discussed in the text and must be corrected or justified prior to any publication decision.The Fornell-Larcker criterion postulates that the square root of the AVE (Average Variance Extracted) of each construct must be greater than any latent correlation between that construct and the others. When cross-referencing the data from Table 8, we observe the following scenario:The square root of the AVE for the Emotional dimension (0.571) is substantially lower than its latent correlation with the Social dimension ($r = 0.669$) and with the Cognitive dimension ($r = 0.678$). Furthermore, the square root of the AVE for the Social dimension (0.667) is also slightly lower than its correlation with the Emotional dimension ($r = 0.669$).The factors share more variance with each other than with their own empirical indicators. The author needs to explicitly justify this in the discussion. A plausible justification is that the specification of a second-order model (where a general Digital Well-Being factor explains the first-order dimensions) assumes and absorbs this high latent correlation.Clarity of Language and Standard English WritingThe English in the text is excellent, fluid, and academic. There is no need for revision by specialized editing services. However, I suggest a brief proofreading of the text.The article presents solid methodological contributions to the assessment of digital behavior and mental health in the Middle East. It features clear writing, a robust ethics committee approval, and open data. However, the author failed to critically report the problems with average variance extracted (AVE) and the violation of latent discriminant validity between the Emotional and Social factors in Sample 2.

Reviewer #4: The manuscript present adequate data and interpretations to corroborate their findings, and discuss a relevant topic. I only have a few suggestions:

- In the description of the scales, add an item representing each factor just as an example

- Why was the maximum likelihood (ML) estimator used? For this sample size, ULS and DWLS were more suited.

**********

7. PLOS authors have the option to publish the peer review history of their article (what does this mean?). If published, this will include your full peer review and any attached files.

If you choose “no”, your identity will remain anonymous but your review may still be made public.

**Do you want your identity to be public for this peer review?**  For information about this choice, including consent withdrawal, please see our Privacy Policy.

Reviewer #3: No

Reviewer #4: No

**********

[NOTE: If reviewer comments were submitted as an attachment file, they will be attached to this email and accessible via the submission site. Please log into your account, locate the manuscript record, and check for the action link "View Attachments". If this link does not appear, there are no attachment files.]

To ensure your figures meet our technical requirements, please review our figure guidelines: https://journals.plos.org/plosone/s/figures

You may also use PLOS’s free figure tool, NAAS, to help you prepare publication quality figures: https://journals.plos.org/plosone/s/figures#loc-tools-for-figure-preparation.

NAAS will assess whether your figures meet our technical requirements by comparing each figure against our figure specifications.

Attachment Submitted filename: PONE-D-26-11267_reviewer.pdf

# Author comment: Author response to Decision Letter 2

**DOI:** 10.1371/journal.pone.0354474.r004

24 Jun 2026

Dear Editorial Office,

Please find attached the revised manuscript, the marked-up manuscript with changes highlighted, and the response to reviewers. The revision addresses the remaining reviewer comments regarding AVE/Fornell–Larcker reporting, example items for each PDWS domain, and the rationale for ML estimation.

Kind regards,

Emadeldin M. Elsokkary

Attachment Submitted filename: PDWS_Response_to_Reviewers.docx

# Editor report: Decision Letter 2

**DOI:** 10.1371/journal.pone.0354474.r005

**Authors:** Tailson Mariano

9 Jul 2026

Psychometric properties of the Arabic Perceived Digital Well-Being Scale (PDWS) in Saudi young adults

PONE-D-26-11267R2

Dear Dr. Elsokkary,

We’re pleased to inform you that your manuscript has been judged scientifically suitable for publication and will be formally accepted for publication once it meets all outstanding technical requirements.

Within one week, you’ll receive an e-mail detailing the required amendments. When these have been addressed, you’ll receive a formal acceptance letter and your manuscript will be scheduled for publication.

An invoice will be generated when your article is formally accepted. Please note, if your institution has a publishing partnership with PLOS and your article meets the relevant criteria, all or part of your publication costs will be covered. Please make sure your user information is up-to-date by logging into Editorial Manager at Editorial Manager® and clicking the ‘Update My Information' link at the top of the page. For questions related to billing, please contact billing support.

If your institution or institutions have a press office, please notify them about your upcoming paper to help maximize its impact. If they’ll be preparing press materials, please inform our press team as soon as possible -- no later than 48 hours after receiving the formal acceptance. Your manuscript will remain under strict press embargo until 2 pm Eastern Time on the date of publication. For more information, please contact onepress@plos.org.

Kind regards,

Tailson Evangelista Mariano, Ph.D.

Academic Editor

PLOS One

Additional Editor Comments (optional):

Reviewers' comments:

# Editor report: Acceptance letter

**DOI:** 10.1371/journal.pone.0354474.r006

**Authors:** Tailson Mariano

PONE-D-26-11267R2

PLOS One

Dear Dr. Elsokkary,

I'm pleased to inform you that your manuscript has been deemed suitable for publication in PLOS One. Congratulations! Your manuscript is now being handed over to our production team.

At this stage, our production department will prepare your paper for publication. This includes ensuring the following:

* All references, tables, and figures are properly cited

* All relevant supporting information is included in the manuscript submission,

* There are no issues that prevent the paper from being properly typeset

You will receive further instructions from the production team, including instructions on how to review your proof when it is ready. Please keep in mind that we are working through a large volume of accepted articles, so please give us a few days to review your paper and let you know the next and final steps.

Lastly, if your institution or institutions have a press office, please let them know about your upcoming paper now to help maximize its impact. If they'll be preparing press materials, please inform our press team within the next 48 hours. Your manuscript will remain under strict press embargo until 2 pm Eastern Time on the date of publication. For more information, please contact onepress@plos.org.

You will receive an invoice from PLOS for your publication fee after your manuscript has reached the completed accept phase. If you receive an email requesting payment before acceptance or for any other service, this may be a phishing scheme. Learn how to identify phishing emails and protect your accounts at https://explore.plos.org/phishing.

If we can help with anything else, please email us at customercare@plos.org.

Thank you for submitting your work to PLOS ONE and supporting open access.

Kind regards,

PLOS ONE Editorial Office Staff

on behalf of

Dr. Tailson Evangelista Mariano

Academic Editor

PLOS One
